# Effect of Periodontal Interventions on Characteristics of the Periodontal Microbial Profile: A Systematic Review and Meta-Analysis

**DOI:** 10.3390/microorganisms10081582

**Published:** 2022-08-05

**Authors:** Sonia Nath, Shaju Jacob Pulikkotil, Laura Weyrich, Peter Zilm, Kostas Kapellas, Lisa Jamieson

**Affiliations:** 1 Australian Research Centre for Population Oral Health, School of Dentistry, Adelaide Health & Medical Sciences Building, The University of Adelaide, Adelaide, SA 5005, Australia; 2 Division of Clinical Dentistry, School of Dentistry, International Medical University, Bukit Jalil, Kuala Lumpur 57000, Malaysia; 3 Department of Anthropology and the Huck Institutes of the Life Sciences, The Pennsylvania State University, University Park, PA 16801, USA; 4 Oral Microbiology Laboratory, Adelaide Dental School, The University of Adelaide, Adelaide, SA 5005, Australia

**Keywords:** 16S rRNA, gene amplification, metagenomics, microbiota, periodontal diseases

## Abstract

Our systematic review aimed to evaluate the effect of periodontal interventions on the diversity and composition of periodontal microbiota assessed by high throughput sequencing (HTS) metagenomics analysis. An electronic search was conducted from database inception to November 2021. All clinical trials that evaluated the effect of periodontal interventions on the gingival microbiota through HTS were selected. The measures of alpha diversity, richness, Shannon diversity index, and the Chao1 index, were used as the primary outcome, whereas relative abundances of bacterial genera were considered as the secondary outcome. Overall, 24 studies were eligible for the systematic review, of which 13 studies were included in the meta-analysis. Periodontal intervention for the test group decreased Shannon diversity, richness, and Chao1 index (alpha diversity), as observed from baseline to post-treatment. The most common genera that increased after periodontal therapy were *Rothia*, *Actinomyces*, *Streptococcus*, *Veillonella*, and *Hemophilus*, whilst *Porphyromonas*, *Tannerella*, *Fusobacterium*, and *Treponema* decreased after periodontal therapy. Periodontal interventions may decrease the bacterial diversity and richness and alter the composition of oral microbiota in the short term. Periodontal microbiota signatures could potentially be used for the assessment of periodontal disease development, progression, and success of the intervention.

## 1. Introduction

The human body is a superorganism with trillions of associated microorganisms that are essential for maintaining health or eliciting disease. The term microbiota generally refers to all organisms comprising bacteria, fungi, protozoa, and viruses, and a perturbation of the healthy microbiota due to complex interactions of genetic, microbial, host, and environmental factors results in the emergence of pathobionts [1]. These microbiota easily outnumber the number of human cells within the body [2]. The total mass of bacteria in an average human is estimated to be about 0.2 kg, and the total number of bacteria cells is around 3.8 × 10^13^ [3]. The activity of the microbiota and the expression of their genomic information, known as the microbiome, provides humans with traits that are not usually present within the human genome [4]. In the mouth alone, over 700 bacterial species have been identified in oral samples by DNA-based microbiome analysis, and they form complex mixtures of species in different micro-niches on the teeth, tongue, and soft and hard tissues of the mouth [5].

Periodontal diseases, including gingivitis and periodontitis, result from inflammation caused by ecological disturbances in the periodontal microbiota [6,7]. In periodontal health, the prevalence of potentially virulent species (pathobionts) are present, but in lower abundance than in individuals with the disease. Clinical evidence suggests that increased periodontal inflammation may not be associated with distinct microbiota, but with an increase in abundance of potentially virulent species, such as *Porphyromonas gingivalis*, *Treponema denticola*, and *Tannerella forsythia*, as well as others, which increases the total bacterial biomass [8]. As a result, several authors have reported increased bacterial richness, evenness, and diversity associated with periodontal disease, although this is unusual, as a disease or microbiota dysbiosis is usually associated with a less diverse microbiome and an increase in certain interventions in other parts of the body [8].

Periodontal treatment consists of a broad range of interventions aimed at controlling the infection and arresting the inflammation [9]. The first step in periodontal therapy consists of non-surgical therapy, i.e., scaling and root planning (SRP) and controlling of risk factors (i.e., smoking and uncontrolled Type II diabetes) [10]. Over the years, researchers have shown that SRP led to a clear improvement in periodontal pocket depth, suppressed periodontal bleeding, and reduced microbial dysbiosis [11]. Several authors have suggested the use of adjuncts to SRP such as antibiotics (commonly amoxicillin and/or metronidazole), mouthwashes (e.g., chlorhexidine), local drug delivery (e.g., doxycycline), and host modulating therapy (e.g., sub-antimicrobial dose doxycycline) [12], especially in periodontitis patients. Periodontal interventions, such as SRP without chemical adjuncts, aim at tampering with the periodontal microflora that would eventually lead to the resolution of inflammation. But these procedures can decrease microbial diversity and significantly reduce the relative abundances of both healthy- and gingivitis-associated bacterial species [13,14]. The development of emerging techniques, such as the potential for oral microbiota transplantation, has also sparked an interest in the response of periodontal microbiota to different therapies to re-establish a healthy microbiota without reducing the bacterial richness and diversity [15]. Novel kinds of toothpaste are also available, which claim to shape the oral microbiota via proteins designed to foster species associated with the healthy oral microbial communities [16]. Similarly, mouthwashes are also available in the market to reduce the bacterial load by antimicrobial effects by altering the plaque microbiota [17,18].

**Figure 1:**
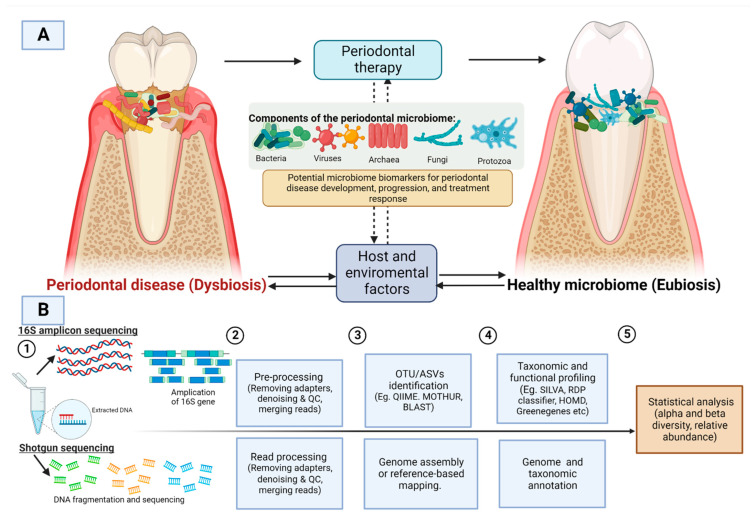
(**A**) Dysbiosis caused by the periodontal microbiome is influenced by various host and environmental factors. Periodontal intervention could alter the microbial composition from a state of dysbiosis (periodontal disease) to eubiosis (periodontally healthy) (**B**) A general workflow for 16S amplicon sequencing and shotgun sequencing. QC: quality control, OTU: operational taxonomic unit; ASV: Amplicon sequence variants. Image created with BioRender.com, accessed om 27 July 2022.

Previous studies have used low throughput measurement techniques (microbial culture, checkerboard hybridization, and PCR analysis), which have resulted in the incomplete characterization of the oral microbiome composition [19,20,21]. Little is known about how the periodontal microbiota composition and diversity changes metagenomically in response to the periodontal treatment [22]. Research can now examine differences in the periodontal microbiota between health and disease through high throughput sequencing (HTS) or next-generation sequencing (NGS). The 16S rRNA amplicon sequencing and random shotgun sequencing are the two main approaches to sequencing. The amplicon sequencing of 16S rRNA genes allows thousands of sequences per sample and provides the power to comprehensively study bacterial community diversity and composition within a specific niche. The shotgun approach allows the study of the entire genome based on random fragments of DNA that are then assembled by finding overlapping ends [23]. There are several bioinformatics tools that provide pipelines used for the generation, clustering, and assigning of operational taxonomic unit (OTU) and building OTU tables and analysing microbial communities and diversities. Recently, with the increased use of denoising methods, amplicon sequence variants (ASVs) are produced instead of clusters. The two most used matrices to assess and compare microbial communities are alpha (within-sample) and beta (between-sample) diversity. The measures of alpha diversity include the number of OTUs/ASVs count or richness (total number of organisms within a sample), the evenness (the relative abundance of the organisms), or indices that combine these two dimensions (e.g., Shannon’s diversity; Chao1 index) [24]. The beta diversity is the number of species shared between microbial communities between samples. The measures of beta diversity include UniFrac, Bray Curtis dissimilarity, Jaccard distance, Principal Component Analysis (PCA), and Principal Coordinates Analysis (PCoA) [25] (Figure 1).

To our knowledge, there has been no meta-analysis conducted on the changes in the periodontal microbiome analyzed through HTS exclusively after the periodontal intervention on periodontal disease patients. This review will be a recent update of the periodontal microbiome literature. The research question was based on the PICOS (Population, Intervention, Comparison, Outcome, and Study) format. In clinical trials (S), we sought to answer: does any form of periodontal treatment (I) compared to intervention without active agent/placebo/SRP (C) affect the composition of the periodontal microbiota (O) involving adults with periodontal disease (P) using HTS methodologies? The authors hypothesized that periodontal intervention could reverse the dysbiotic microbiota associated with periodontal disease towards a balanced state consistent with oral health.

## 2. Materials and Methods

### 2.1. Study Registration

The study review protocol was registered with the PROSPERO database (International Prospective Register of Systematic Reviews), registered under CRD42020188531, and can be accessed on https://www.crd.york.ac.uk/prospero/display_record.php?RecordID=188531. This systematic review and meta-analysis has been prepared according to the Preferred Reporting Items for Systematic Reviews and Meta-Analysis (PRISMA) guidelines [26].

### 2.2. Specific Research Goals

1. To find the changes in the composition of the periodontal microbiome community after periodontal treatment. By composition, we intend to find patterns in disease-associated shifts in the periodontal microbiota that differ in their directionality (microbiota pre-intervention vs. microbiota post-intervention);
2. To find the magnitude of difference in alpha diversity metrics before and after periodontal treatment. Some of the commonly used measures include several OTU counts (richness), Shannon diversity index (accounts for both abundance and evenness of the species), and Chao1 index (non-parametric method for assessing the number of species in a community). To find the difference in beta diversity before and after treatment. The commonly used measures of beta diversity include UniFrac, PCA, PCoA, Bray Curtis dissimilarity, Jaccard distance, and Principal Coordinates Analysis; and
3. To find the predominant bacterial species present in the periodontal microbiome and the total number of bacterial species that differ between the treatment group and control group identified through high throughput sequencing.

### 2.3. Search Strategy

An electronic search was conducted on the following databases to identify eligible studies: MEDLINE, Scopus, and EBSCOHost (Dentistry & Oral Sciences Sources). For grey literature, we used the Cochrane database of systematic review, OpenGrey database, ProQuest Dissertation, and clinicaltrial.gov. In addition, a manual hand-searching of reference lists of relevant papers was screened to identify articles that might have been missed on the electronic search. We refrained from using Google Scholar; although it is a very powerful tool, it has a low threshold of reproducibility, accepts only very basic Boolean logic, the algorithm by which the search results are ordered has not been disclosed, and it may not be an effective means of identifying grey literature [27]. The articles were searched from database inception to November 2021.

The search strategy used for the MEDLINE database was (“Metagenomics” [MeSH] OR “Metagenome” [MeSH] OR “High-Throughput Nucleotide Sequencing” [MeSH] OR “microbiota” [MeSH] OR “Genes, Bacterial” [MeSH] OR “metagenomics” [tiab] OR “16S rDNA” [tiab] OR “16S rRNA” [tiab] Or Pyrosequencing [tiab] OR “next-generation sequencing” [tiab] OR “Illumina sequencing” [tiab] OR “Functional gene array” [tiab] OR “Oral microbiome” [tiab] OR “Bacteria*” [tiab] OR “Bacterial diversity” [tiab] OR “Bacterial community” [tiab]) AND (“Tooth Diseases” [MeSH] OR “Mouth Diseases” [MeSH] OR “Oral Health” [MeSH] OR “Gingival diseases” [MeSH] OR “Gingivitis” [MeSH] OR “Periodontal diseases” [MeSH] OR “Periodontal debridement” [MeSH] OR “Periodontal index” [MeSH] OR “Periodontal pocket” [MeSH] OR “probing depth” [tiab] OR “periodont*” OR “plaque score”).

For Scopus and EBSCOHost, we used a similar search strategy, but for our grey literature search, we used a truncated search string to maximize the number of results (Table A1). The search string was pilot tested using a combination of MeSH and key terms. Systematic reviews, narrative reviews, and standard textbooks were additionally searched to identify all the other eligible studies. Whenever possible, citation tracking was performed on search engines to keep track of the latest publication.

### 2.4. Study Selection Criteria

#### 2.4.1. Types of Participants

The population selected was any individual ≥18 years of age who underwent any procedure or intervention for restoration of periodontal health. As we get older, the oral microbiome is stabilized compared to that of youth or children. This is due to the establishment of independent oral hygiene, maintenance habits, permanent dentition, and a stable adult diet with defined dietary patterns [28,29]. Having said that, even the adult microbiome can be altered throughout life with changes in dietary habits, increasing age, oral hygiene practices, and tobacco and alcohol use [29,30].

#### 2.4.2. Type of Interventions

Interventions aimed at achieving periodontal health were included. All types of interventions such as standard periodontal therapy (i.e., SRP either as the sole procedure (having controls as no treatment) or combined with mouthwash or antibiotics, toothpaste with active agents, and customized diets for achieving periodontal health) were included. Standard treatment, including SRP, antibiotics (amoxicillin, or metronidazole), toothpaste without active ingredients, placebos, or no treatment, were included as controls. For both test and control groups, changes in microbial communities were noted at baseline and post-intervention. Therefore, we are comparing: (1) test vs. control and (2) each group individually (test group (baseline vs. post-intervention) and control group (baseline vs. post-intervention)). Trials that evaluated the effectiveness as a single interventional trial arm by comparing before and after treatment (baseline vs. post-intervention only) were also included, though there was no control group for comparison.

#### 2.4.3. Types of Outcome Measures

The primary outcomes were measures of (1) alpha diversity of periodontal microbiota at baseline compared to post-treatment values for treatment and control subgroups (mean ± standard deviation) and (2) comparison of alpha diversity among treatment and control group post periodontal intervention (mean ± standard deviation). The relative abundance level (relative prevalence percentage) for each bacterial genera and species and beta diversity were included as secondary outcomes.

### 2.5. Selection Criteria

#### 2.5.1. Inclusion Criteria

Studies with the following criteria were selected: (1) original studies; (2) evaluation of an intervention compared to control for restoration of periodontal health; (3) having periodontal disease (gingivitis and periodontitis); (4) analysis of periodontal microbiota through bacterial high throughput sequencing; and (5) randomized clinical trial, before and after trials and/or quasi-experimental.

#### 2.5.2. Exclusion Criteria

Studies that analyzed salivary microbiome, tongue scraping, or other parts of oral mucosa instead of supragingival plaque or subgingival plaque for microbial profiling were excluded. Healthy participants without any forms of periodontal disease were excluded. In vitro or animal studies, case reports, case series, retrospective studies, literature reviews, opinion papers, letters to conference proceedings, and abstracts were also excluded.

#### 2.5.3. Selection of Studies

All the articles found through searching of the databases were uploaded in referencing software, EndNote X9 Version 3.3 (Clarivate Analytics, Philadelphia, PA, USA), and the duplicates were removed. All the references were imported into systematic review management software, Covidence, and were used for screening title and abstracts and full texts. Two independent and calibrated reviewers (SN and SJP) assessed the studies by title and abstracts against the eligibility criteria. The full text was uploaded in Covidence, and assessments were performed only for selected articles that followed the inclusion and exclusion criteria. If there was any disagreement and consensus could not be reached among the two reviewers, a third reviewer (LMJ) was referred for the final decision. The reasons for the exclusion of an article have been recorded separately.

### 2.6. Data Extraction

Data extraction was performed independently from the selected studies by the two reviewers (SN and SJP) using a customized data extraction form containing all information necessary to answer the research question (Table A2). The data extraction form was first pilot tested on a random sample (5% of included studies) and modified until all the reviewers agreed upon the key variables and outcomes. There are various computational diversity matrices used among researchers for assessing alpha diversity such as Pielou’s evenness index, observed ASVs and OTUs count, Faith’s phylogenetic diversity, etc. However, for our review, the three most used (richness (including ASVs/OUT count), Shannon index, and Chao1 index) were selected as outcomes. When the included studies had any missing information, the authors were contacted via email correspondence for further details and information. The data extraction form included details on (1) study characteristics: surname of the first author, year and country of study, study design; (2) patient characteristics: disease type and definition, age, number of participants for test and control group, treatment and control description, and duration of treatment; (3) collection and extraction method: plaque collection method and hypervariable region for sequencing; and (4) outcome measures: Alpha diversity (richness, Shannon diversity, and Chao1 index) and relative abundance levels for genera identified in the study. The method of OTU/ASV generation, databases used taxonomic and functional profiling, and methods used for statistical analysis has been recorded for each included publication.

For pooled data and meta-analysis, data extraction from graphs or charts was performed using the WebPlotDigitizer tool (Version 4.2 GNU Affero General Public License). In studies that reported only the median, the estimates were converted to mean and standard deviation [31].

### 2.7. Assessment of Risk of Bias and Quality of Evidence Assessment

For quality assessment of clinical trials, the “Risk of Bias Assessment 2” (RoB 2.0) guidelines formed by the Cochrane Collaboration was used [32]. For each study, bias was assessed in five domains: (1) bias due to randomization; (2) bias due to deviations from intended intervention; (3) bias due to missing data; (4) bias due to outcome measurement and (5) bias due to selection of reported results. Each clinical trial was scored as ‘high risk’, ‘some concerns’, or ‘low risk’ and given an overall score. The robvis visualization tool was used for visualizing the risk of bias assessment. No study would be excluded based on the quality of the paper.

The quality of evidence across the included studies was assessed using the Grading of Recommendations Assessment, Development and Evaluation (GRADE) methodology [33]. The risk of bias and quality of evidence assessment was performed independently and in duplicates by two reviewers (SN and SJP). Any disagreements were resolved through discussion between the two reviewers, or a third reviewer was consulted (LMJ).

### 2.8. Statistical Analysis

All data were extracted and collated in a spreadsheet. All the findings are represented in narrative table form, and where possible, the data was meta-analyzed. Meta-analysis was performed using a random-effects model. We used the random-effects model in contrast to the fixed-effect model, as the former model assumes that the observed estimates of treatment can vary across studies due to real differences in the interventions and sampling variability [34]. In our review, we expected heterogeneity in periodontal interventions, study population, and follow-up length. We used the restricted maximum likelihood (REML) method as the estimation method for meta-analysis, which is a commonly used method when the number of studies is small and produces an unbiased estimate of between studies variability [35]. To compute an effect size, Hedges’s *g* standardized mean difference and 95% confidence intervals were estimated for measures of alpha diversity (1) within a group test (baseline vs. post-treatment) and control groups (baseline vs. post-treatment) and (2) between groups (test vs. control group). Heterogeneity was measured using Galbraith plots and I^2^ statistics. A galbraith plot is a scatterplot of the standardized effect size, used as an alternative to a forest plot for assessing heterogeneity and detecting potential outliers. I^2^ was used to represent the percentage of variation attributable to statistical heterogeneity and was categorized as low (25–50%), moderate (51–75%), or high (>75%) [36]. The leave-one-out meta-analysis performs multiple meta-analyses, omitting one study each time, such as sensitivity analysis. There is a tendency for smaller studies to report larger effect sizes than larger studies, which could be due to between-study heterogeneity and publication bias. Publication bias was assessed using contour-enhanced funnel plots constructed for visualization. Asymmetry in the plots may indicate publication bias. The meta-analysis was performed using STATA 17 software (StataCorp. 2017, Stata Statistical Software: Release 17.0 StataCorp LLC, College Station, TX, USA).

### 2.9. Power Calculation for Meta-Analysis

We performed a power calculation that allowed us to assess whether the included studies had sufficient statistical power to detect small effect sizes. Power calculation was carried out according to the methods described by Bohrentein et al. (2009) [37]. Since there was no previous meta-analysis performed, we conducted the calculation based on the effect size (*d* = −0.60) we calculated in our meta-analysis (alpha diversity between treatment and control group) and found that at least 7 studies are required with 200 participants for the test and control group assuming high heterogeneity and statistical power of 80% and alpha of 5%.

## 3. Results

### 3.1. Search Strategy and Screening Process

**Figure 2:**
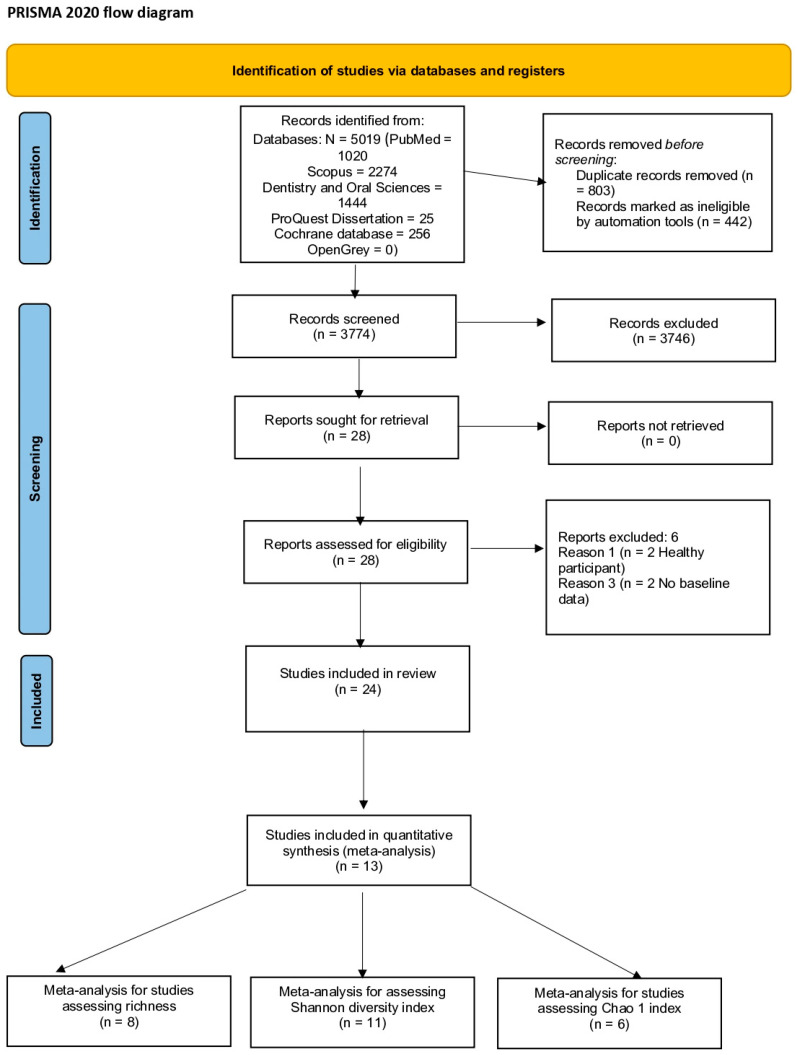
PRISMA flow chart 2020 of the included trials.

The search details are provided in the PRISMA flow chart (Figure 2). The search strategy resulted in 5019 potential articles: 1020 articles from PubMed (MEDLINE), 2274 articles from Scopus, 1444 articles from the Dentistry and Oral Sciences database, 25 from ProQuest Dissertation, 256 from Cochrane database of systematic reviews, and no articles from OpenGrey. After the removal of 1245 articles as duplicates, 3374 articles were screened. All articles were assessed according to the inclusion and exclusion criteria, and this led to a full-text analysis of 28 articles [6,13,14,16,17,18,23,38,39,40,41,42,43,44,45,46,47,48,49,50,51,52,53,54,55,56,57,58]. Two studies did not report baseline data [6,53], and two studies assessed healthy participants [16,46]; therefore, all four were excluded. Overall, 24 studies were included for descriptive analysis and 13 studies [13,14,17,18,39,41,43,44,49,51,52,57] were meta-analyzed.

### 3.2. Characteristics of Included Studies

Characteristics of the included studies were examined in the context of study design, periodontal disease definition, age, description of test and control group, plaque collection method, hypervariable region of 16S rRNA gene, and follow-up period (Table 1).

#### 3.2.1. Study Type and Intervention

Of the 24 studies, 14 were randomized clinical trials (RCTs) [13,17,18,39,41,43,44,47,48,49,51,54,56,57], whereas eight studies [14,23,38,42,45,50,55,58] were pre- and post-intervention studies (before and after SRP) and two studies [40,52]) were clinical trials without clearly defining randomization. For this analysis, one RCT [13] was split into three sub-studies, two prevention sub-studies and one treatment sub-study, while another clinical trial [39] described their results as above or below median responses; thus, we analyzed this data separately. In total, six types of interventions were observed: (a) seven studies [14,23,38,42,45,55,58] were single-arm studies comparing pre- and post-SRP; (b) five studies supplemented SRP with antibiotics [39,43,48,50,51] or probiotics [41]; (c) four studies [13,18,40,56] used mouthwash rinses only; (d) two studies [49,52] compared ultrasonic scaling to air polishing; (e) three studies [17,44,47] used toothpaste with active ingredients; (f) one study used a regenerative material (Beta-tricalcium phosphate/EMD/Hydroxyapatite) for furcation therapy [55]; and (g) one study used an anti-inflammatory diet [57]. The duration of the interventions ranged from immediately after treatment [58] to three weeks [13,18], 4 weeks [17,41,42,45], 6 weeks [14,55,57], 8 weeks [43,48,50], 12 weeks [38,40,44,47,49,52], 6 months [51,54,56], and 12 months [39].

#### 3.2.2. Study Participants

All the study subjects were generally systematically healthy patients, and the age ranged from 18–73 years. All the studies included both the male and female sex except for one study [45] that only included female patients. For this review, we included studies on all forms of periodontal diseases, including generalized gingivitis [6,55,57], chronic periodontitis [23,38,39,42,43,44,48,49,54,55,56], aggressive periodontitis [14,45,50], periodontitis (unclassified) [40,41,47,52,58] and experimental gingivitis [13,18].

#### 3.2.3. Study Methodology and Metagenomics Analysis

The plaque collection methods varied across studies; the most popular methods for collection of plaque was sterile paper points [13,39,42,43,44,48,49,50,54,56] and sterile Gracey curettes [17,18,23,38,40,41,45,51,52,57,58] or periodontal scaler [55]. Few authors preferred filter paper [14] and swabs [47] for plaque collection. The most commonly used hypervariable region observed was the V3–V4/ V4 [14,38,42,43,45,50,51,52,56,57] region of 16S rRNA, while some authors used V1–V2 [13,17,18,55,58], V4-V6 [44], V4–V5 [40,47], V5–V7 [39], and V6 [48].

#### 3.2.4. Bioinformatics and Statistical Test

The most popular method for sequencing is 16S rRNA sequencing (Table 2); however, one author used a combination of shotgun and 16S amplicon sequencing [40] and one author used only shotgun sequencing [23]. The OTU-based method for clustering has been commonly used across many of the studies, i.e., more than 97% similarity. All the studies used OTU as the basis of metagenomics analysis, except for two studies [43,44] that used ribosomal sequencing variants (RSVs), and two authors used amplicon sequence variant (ASV) [47,51]. The commonly used platforms for generating OTU/abundance tables from raw sequence reads were MOTHUR or QIIME/QIIME 2 (Quantitative Insights into Microbial Ecology) or pipelines in R programming software (DADA2, Phyloseq) used independently or in comparison with a referencing database such as Human Oral Microbiome database, SILVA 16S rRNA database, Greengenes database, and RDP.

Additional statistical analysis was carried out by several authors for hypothesis testing of alpha and beta diversity indices before and after periodontal therapy. Depending on the normality and non-normality of the data, either *t*-test [23,49,52,58], analysis of variance (ANOVA), or a non-parametric test such as Wilcoxon rank-sum/signed signed-rank test [13,18,41,44,52,56,58] or Mann–Whitney test [13,14,39,41,44] and Kruskal Wallis test [38,57] was performed. Categorical variables were analyzed using the Chi-squared test, and McNemar and Fischer test [41]. For analyzing the association of microbial composition and environmental covariates and outcomes, several multivariate analysis methods were used. The analysis of group similarities (ANOSIM) [14,23,47], analysis of covariance (ANCOVA) [17,39,44], and multivariate analysis of variance was conducted with permutation (PERMANOVA) [39] and the Mantel test [40]. Sample size calculation was only carried out by few clinical trials [17,41,47,51,54,56,57].

#### 3.2.5. Measures of Alpha Diversity

Alpha diversity is a measure of within-sample diversity of the community, described in terms of the number (richness) or distribution (evenness) [24]. The bacterial richness (number of observed species) was assessed commonly by most authors [13,14,18,43,44,47,49,58]. The commonly used indice to measure alpha diversity was Shannon Diversity Index [13,14,17,18,39,41,43,51,52,58]. Some studies used the Chao1 index [13,14,47,51,52,58], Simpson index [14,42,48], Pilous evenness [43,48], and Faith’s phylogenetic diversity [40]. The findings of alpha diversity from the included studies have been described in Table 2.

#### 3.2.6. The Measure of Beta Diversity

Beta diversity is a measure of the between-sample differences between pairs of communities. It can be measured either using principal coordinates analysis (PCoA) or principal component analysis (PCA) [25]. To assess the beta-diversity, PCoA was used in twelve studies [13,14,17,23,40,43,44,45,47,51,52], and only three studies used the PCA [18,39,57]. Meta-analysis could not be conducted for beta diversity. The descriptive finding of each included study is summarized in Table 2. We tried contacting the authors of the papers for raw primary data for beta-diversity for meta-analysis but failed to receive any additional information.

#### 3.2.7. Relative Abundance of Bacterial Genera

**Figure 3:**
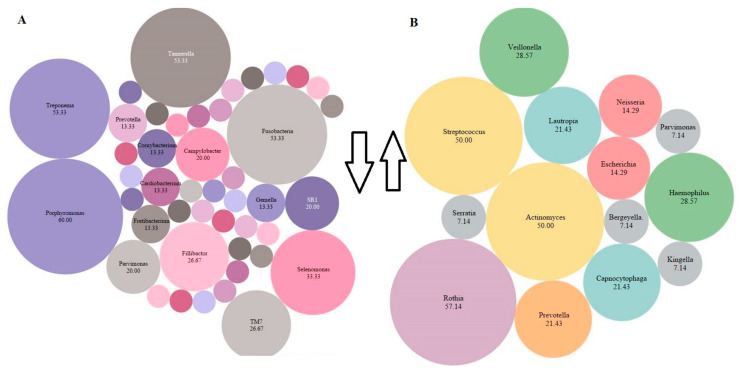
The most abundant species present across all the studies post-intervention. The percentage of abundance was calculated by cumulative % of all bacterial species present in the included articles. The size of the bubble is related to the abundance (higher abundance = large bubble). The smaller bubbles represent species <7% (*Megasphaera*, *Oribacterium*, *Peptococcus*, *Peptostreptococcus, Solobacterium*, *Actinomyces*, *Atopobium, Bacteroidacea*, *Clostridiales*, *Cornynebacterium*, *Dialaster*, *Granulicatella*, *Kingella*, *Mogibacterium*, *Olsnella*, *Propionibacterium*, *Spirochates*, *Stretococcus, Veillonella*. (**A**) Decrease (downward arrow) in species post-intervention (**B**) Increase (upward arrow) in species post-intervention. The drawing was made in Tableau version 9.1, Seattle, WA, USA.

One of the main findings of the review was the consistency of the identified bacteria among the studies. We tried to identify the most abundant species before and after periodontal intervention and tried to find the common species that decreased and increased after periodontal intervention (Table A3). There was more prevalence of bacteria of the Red complex in the pre-intervention stage and more health-associated bacteria after an intervention. Post-intervention *Porphyromonas*, *Fusobacterium*, *Tannerella*, *Treponema*, *Selenomonas*, *Parvimonas*, TM7, *Fillibactor*, *Fretibacterium*, and *Campylobacter* decreased in number. While *Escherichia*, *Neisseria*, *Prevotella*, *Capnocytophaga*, *Lautropia*, *Haemophilus*, *Veillonella*, *Actinomyces*, *Streptococcus*, and *Rothia* increased in number post-intervention (Figure 3).

### 3.3. Synthesis of Results

#### 3.3.1. Within-Group Alpha Diversity for Treatment and Control Groups (Baseline vs. Treatment)

**Figure 4:**
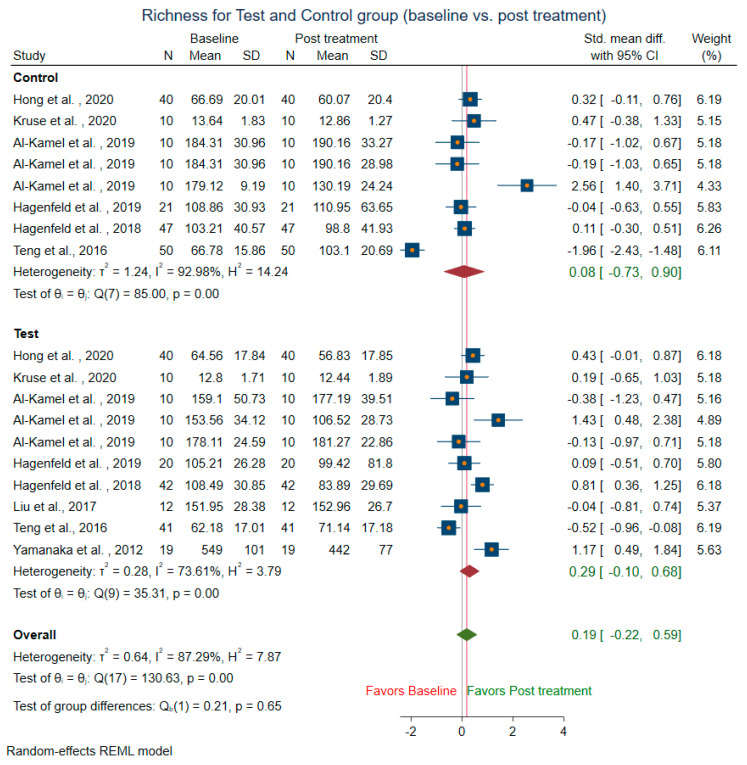
Subgroup meta-analysis of richness based on test subgroup (baseline vs. post-treatment) and control subgroup (baseline vs. post-treatment).

**Figure 5:**
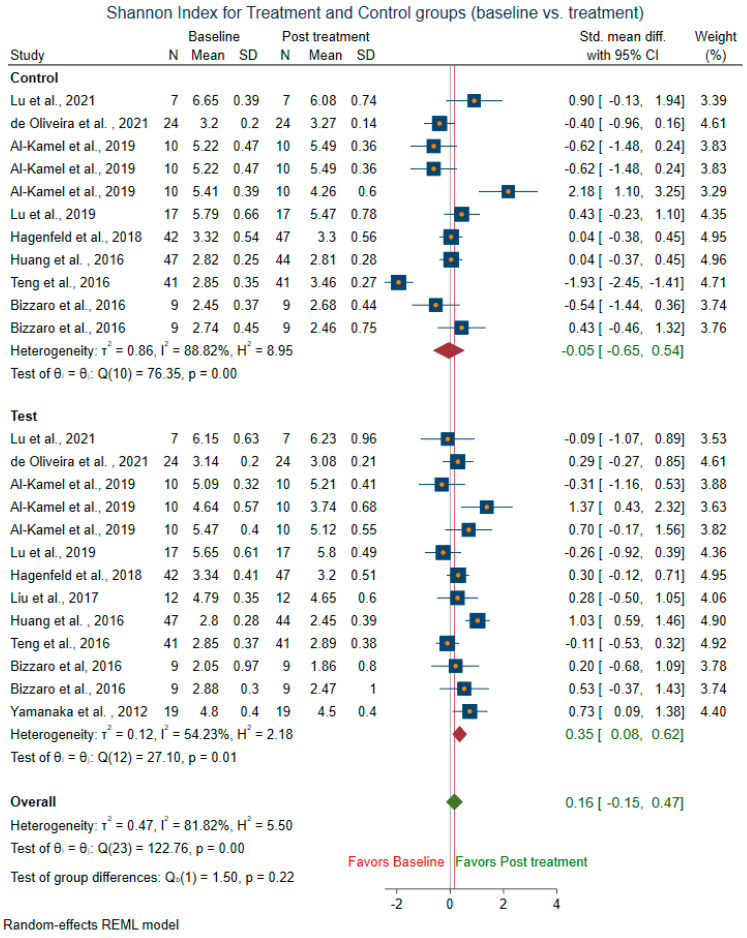
Subgroup meta-analysis of Shannon index based on test subgroup (baseline vs. post-treatment) and control subgroup (baseline vs. post-treatment).

**Figure 6:**
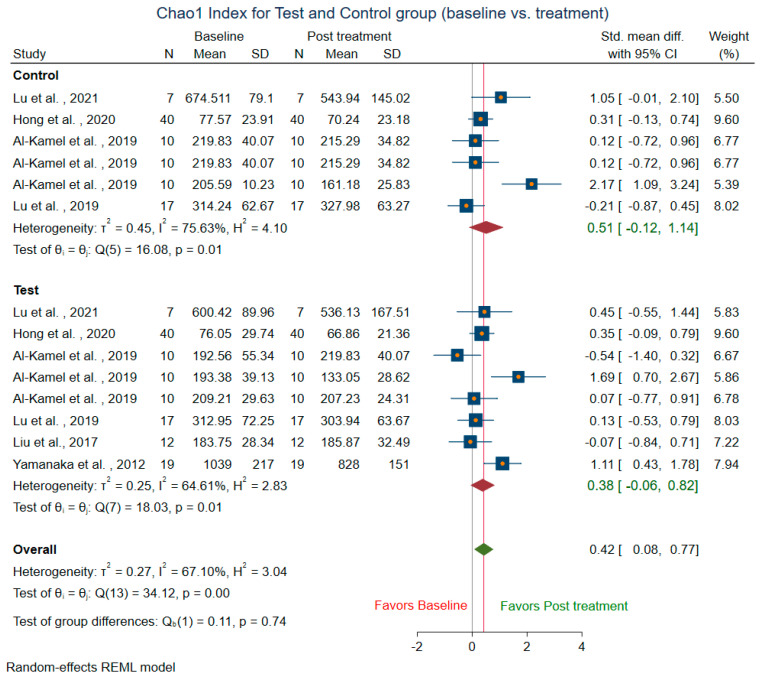
Subgroup meta-analysis of Chao1 index based on test subgroup (baseline vs. post-treatment) and control subgroup (baseline vs. post-treatment).

Meta-analysis could not be conducted for studies that did not include mean and SD. Two studies [14,58] were before and after the trial, did not have control groups, and were excluded from the control sub-group analysis. The difference in alpha diversity from baseline to post-intervention was compared independently for both pooled test and pooled control groups. Post-intervention, the pooled test group showed lowered richness, Shannon’s diversity, and Chao1 index when compared to the control group. Eight trials [13,14,18,43,44,47,49,58] revealed a decrease in richness in the treatment group (SMD = 0.29; 95% CI = −0.10, 0.68) (Figure 4). In contrast, the richness resembled baseline in the control group (SMD = 0.08, 95% CI = −0.73, 0.90). Ten studies for the test group were meta-analyzed for Shannon’s diversity [13,14,17,18,39,41,43,51,52,58] (Figure 5). The test group showed a decrease in Shannon diversity in the post-treatment samples (SMD: 0.35, 95% CI: 0.08, 0.62), whereas the control group (SMD: −0.05, CI: −0.65, 0.54) resembled baseline. Six trials examined the Chao1 of observed species [13,14,47,51,52,58] (Figure 6), and there was a decrease in the Chao1 Index between baseline and post-treatment in the test group (SMD: 0.38, 95% CI: −0.06, 0.82) and the control group (SMD: 0.51, 95% CI: −0.12, 1.14).

#### 3.3.2. Between-Group Alpha Diversity (Test vs. Control)

Two studies [14,58] were dropped from the analysis, as they did not have control groups. The richness was lower after periodontal intervention in the treatment group than in the control group (SMD: −0.45, 95 CI: −1.34, 0.43) (Supplementary Figure S1). Similarly, Shannon index (SMD: −0.61; 95% CI: −1.27, 0.06) (Supplementary Figure S2) and Chao1 index (SMD: −0.18; 95% CI: −1.19, 0.84) (Supplementary Figure S3) was lower among treatment groups when compared against the control group favoring periodontal treatment.

#### 3.3.3. Heterogeneity among Studies and Publication Bias

For detection of heterogeneity, we used I^2^ statistics and Galbraith’s plot. The I^2^ was high (>80%) for all the forest plots of alpha diversity. The Galbraith scatter plot for richness showed all the studies except one lowered the richness after periodontal treatment (all the dot points are below the no-effect line) (Supplementary Figure S4) and heterogeneity was detected; three studies were outliers out of seven studies. For the Shannon index, there were four outliers out of ten groups (Supplementary Figure S5), and for the Chao1 index there were two outliers (Supplementary Figure S6), which may be the reason for heterogeneity.

The leave one out sensitivity analysis showed that one study [13] might have influenced the outcome for richness (Supplementary Figure S7), Shannon index (Supplementary Figure S8), and Chao1 (Supplementary Figure S9).

The contour-enhanced funnel plots were asymmetrical for all the measures of alpha diversity (Supplementary Figures S10–S12), but no small study effect was detected, and no publication bias was detected, as there were studies present in the non-significant region.

### 3.4. Risk of Bias Assessment and Quality of Evidence

The robvis tool was used for visualizing the risk of bias assessment (Supplementary Figure S13). The risk of bias for each study was assessed for all domains, as described in the Cochrane Handbook [32]. From the 24 studies, the overall risk of bias was high for all but six trials [13,41,44,47,49,51]. The main reason for high-risk bias was unclear reporting about random sequence generation and allocation concealment.

The GRADE analysis assessed the evidence of the outcome for alpha diversity (richness, Shannon, and Chao1) between the test and control groups, and they were of low quality (Supplementary Figure S14). This could have been due to a lack of randomization, high heterogeneity among studies, and lack of consistency in the methodology.

## 4. Discussion

To the best of our knowledge, this is the first systematic review that assessed the effects of periodontal interventions on the diversity of periodontal microbiota meta-analytically. The effects of periodontal interventions on the diversity and abundance of certain genera were assessed to test if periodontal interventions could alter the microbial composition of the periodontal plaque to a resolved or healthier state. The findings from our review suggest that periodontal intervention could lead to low diversity, richness, and community evenness, as we observed a decrease in the alpha diversity.

When interpreting the results of this systematic review, the following limitations should be considered. The heterogeneity could be primarily due to methodological variation (study design, disease levels, interventions, and study population) and sequencing techniques. There were a limited number of clinical trials reporting on the periodontal microbiome. We used six databases for our search strategy and could not expand our search strategy to other databases (e.g., Web of Science, Embase), and this could have led to publication bias; publications in the English language were selected, and this could have led to additional bias. For the meta-analysis, we have combined all forms of periodontal intervention which were directed toward treating periodontal disease. The main goal of this review was to observe changes in microbial communities (alpha and beta diversity) before and after periodontal therapy, regardless of the intervention. Therefore, we grouped studies based on the outcome and not the treatment. Previously, authors had similarly combined interventions in meta-analysis for the gut microbiome [59,60]. Periodontal disease includes both conditions, gingivitis, and periodontitis, and for this review, they were not separated. We included studies that have assessed the periodontal microbiome through a sampling of supra and subgingival plaque. Periodontal pathogens found in saliva may not be reflective of periodontal microbiota [38] and were excluded from this review. As observed from our review, the method of plaque collection varied from the use of paper points and sterile Gracey curettes, but both methods are equally effective for the collection of samples [61]. The included studies also used a wide range of different interventions to restore periodontal health from common procedures such as SRP to diet. The patient population also differed in geographic and ethnic variability.

The composition and diversity of the microbiome could be dependent on factors such as the hypervariable region selected, DNA extraction methods, sampling, the sequencing platform used, and the database for taxonomy [62]. The OTUs, ribosomal sequence variants (RSV) or ASVs, the number of reads per sample, the quality of filtering, and the normalization of data could also be the reason for heterogeneity. The authors of this review found methodological inconsistencies for HTS in all the included studies. For example, there were inconsistencies in DNA extraction and library preparation method and variability in marker selection (e.g., V_1_–V_3_ vs V_3_–V_4_). The laboratory environment should be standardized and applied across studies. Our systematic review and meta-analysis included studies with similar methods and outcome measures for microbial profiling. Systematic and comparable methodologies and rigorous statistical analysis are required for a more accurate and precise estimation of these effects. The latest use of HTS of the 16S rRNA gene allows a deeper analysis of the subgingival microbiota, but also has limited our ability to interpret data sets with wide ranges in methodologies, techniques, and analysis methods [63]. We also found most authors used the traditional statistical methods for microbial data analysis. Sparsity with many zeroes, overinflation, and over-dispersed data often poses a challenge for accurate statistical analysis for microbiome researchers. Handling rare taxa, and large *p* and small *n* [64] pose additional statistical issues. Due to these problems, the traditional parametric and non-parametric methods might not be suitable to analyze the microbiome data with many excess zeroes, and failure to account for this may result in misleading inference and estimation.

Periodontal disease leads to an oral microbiota with higher richness, evenness, and diversity. There is a shift in the microbial communities at the species and even at the strain/clone level. A diverse combination of species has been reported, but oral health-associated species are usually lower in frequency [8,65], whereas periodontal health is related to lowered diversity and evenness [23]. Our findings suggest that the richness and diversity were significantly lower in the pooled (all studies) treatment group compared to baseline. Periodontal interventions resulted in a less rich and diverse microbiota, which is a normal characteristic seen in a periodontally healthy individual. Therefore, periodontal interventions might have resulted in a shift in oral microbiota with fewer variations in the microbial community. Our findings are in contrast to those of Galimanas et al. (2014) [66] and Kirst et al. (2015) [67], who failed to capture any differences in microbial diversity and overall composition between healthy (no periodontitis) and periodontitis patients. While an overriding host defence could limit the community composition to non-pathogenic commensals, technical and methodological implications also need to be further investigated when comparing studies [65]. Overall, the diversity depends on the mean number of reads. A lot of information might be lost if the number of reads is too low due to extraction methods, and low abundance OTU might be discarded or the reads might be too short and might not provide a good identification of the microbial community [68,69]. The low abundance of organisms may make all the difference among the studies. NGS is moving towards producing longer reads (>500–700 bp for one single 16S rRNA) and therefore better identification. However, the targeting of the variable regions to the most appropriate region of 16S rRNA gene even, with larger reads, is more crucial. The microbiome is resilient due to richness, kinds of interactions, and functional redundancy.

While there is considerable debate about the presence of a core human microbiota, many microbes are generally common among most individuals and comprise dominant species that exist under healthy conditions [1]. The variable sets of species that are exclusive to an individual are often linked to lifestyle and environmental changes. It additionally depends on phenotypic and genotypic determinants, as well as one’s evolutionary history [70,71]. This signal is as unique across individuals as a human fingerprint [4]. In our review, we found several species increased and decreased post-intervention, and these species could be used as periodontal-based signatures for assessment of the efficacy of the periodontal intervention. The species *Porphyromonas*, *Tannerella*, *Fusobacterium*, *Treponema*, and *Selenomonas* were found consistently to decrease post-intervention among all the pooled studies. Collectively, these species are associated with gingivitis and chronic periodontitis [1,6,13,67]. A decrease in the abundance of these species has been associated with recovery from periodontal disease. *Streptococcus*, *Actinomyces*, *Rothia*, *Veillonella*, *Prevotella*, and *Veillonella* increased post-intervention. Among these, the classes *Firmicutes* (e.g., *Streptococcus*) and *Actinobacteria* (e.g., *Actinomyces*) were most commonly found (Figure 2). Previous studies have shown that *Firmicutes* and *Actinobacteria* are routinely found in dental plaque from healthy individuals [72] and *Streptococcus* have also been associated with healthy oral conditions and are an indicator of periodontal stability [63]. Similarly, *Actinomyces* are generally associated with health [1,6,13]. Due to a limited number of eligible studies, the abundance could not be calculated at a species level, which is a more robust technique to discriminate the role of specific community members in driving the ecological shift [16]. A combination of both amplicon and shotgun sequencing could identify bacteria more in-depth to the species level. The main advantage of HTS is the ability to discriminate species at the clonal level.

The current interventions for periodontal treatment aim at reducing the bacterial biomass but do not necessarily return it to a state of periodontal health [8]. Our review highlights the fact there is not a single composition of bacteria that represents either a healthy state or a diseased state, e.g., the genus *Prevotella* and *Streptococcus* are detected in health and some species are detected in disease. There has been an increase in interest in therapeutic interventions that can restore periodontal health by modulating microbial ecology [73]. Oral microbiome transplantation (OMTs) could be potentially beneficial in replacing diseased microbiota with healthy microbes [15]. This could ensure that the diseased site is repopulated with microbiota associated with health.

## 5. Conclusions

There is a need for more standardized clinical trial and NGS methods and validated reference databases for better taxonomy. Here, we conduct a preliminary, qualitative comparison to begin to understand the overarching trends of how periodontal microbiota respond to treatment.

Decreased alpha diversity indicates a shift of the periodontal microbiota towards periodontal health following a periodontal intervention. Few genera were consistently present in many of the included studies and could be part of the core microbiota. There was consistency in the microbiota species that increased and decreased post-intervention and could serve as microbiota-based signatures for understanding and comparing different periodontal interventions in the future.

### Future Research

Future research in this area should be oriented towards examining different treatments while adopting the same methodologies for oral microbial profiling. Our findings highlight the importance of complete characterization of the periodontal microbiota to accurately evaluate the effectiveness of new therapeutic and diagnostic methods for periodontal disease treatment. The periodontal microbiota could be an alternative target for new therapies and should be monitored to better understand the efficacy of periodontal treatment outcomes.

## Figures and Tables

**Table 1:** Characteristics of included studies according to treatment groups.

First Author and Year Country	Study Design	Disease	Disease Definition	Age	Case (n)	Control (n)	Test Description	Control Description	Site and Plaque Collection Method	Hypervariable Region of 16S rRNA Gene	Follow Up	Outcome
**Scaling only**												
Yamanaka et al., 2012 * [58] Japan	Pre/post-intervention	Periodontitis	N/A	35–73	19		SRP	Before SRP	Supragingival Sterile curettes	V1–V2 region	Immediately	Periodontal therapy decreased microbial richness and biodiversity in the plaque microbiota.
Schwarzberg et al., 2014 [55] Germany	Pre/post-intervention	Gingivitis, mild to moderate or severe periodontitis	Gingivitis: CAL ≤ 3 mm, PD ≤ 4 mm, BOP > 10%; mild-moderate periodontitis: CAL ≥ 4 mm, PD ≥ 5 mm, BOP ≥ 30%; severe periodontitis: CAL ≥ 6 mm, pocket depths ≥ 7 mm, BOP ≥ 30%	21–40	23 (gingivitis); 12 (mild/mod periodontitis; 1 (severe periodontitis)	4	Standard periodontal treatment	N/A	Subgingival plaque Periodontal scaler	V1–V2 region	6 weeks	Individual differences were observed and the relative abundance of disease related bacteria and health relative bacteria showed variability.
Shi et al., 2015 [23] USA	Pre/post-intervention	Chronic periodontitis	N/A	53	17		SRP	Before SRP	Subgingival plaque Sterile Gracey curettes	Metagenomic shotgun sequencies & 16S rRNA was extracted.	4–19 weeks	After SRP there was a decrease in alpha diversity, change in composition towards health-associated bacteria, lowered diseased associated functional pathway, and reduction in microbial correlation.
Liu et al., 2018 * [14] China	Pre/post-intervention	AAP 1999 definition for generalized Aggressive Periodontitis	Rapid attachment loss and bone destruction, and possible familial aggregation of disease.	30.75 ± 3.17	12		SRP	No treatment	Subgingival plaque Filter paper	V3–V4 region	6 weeks	SRP was effective in reducing alpha diversity, changing bacterial compositional structure, reducing the relative abundance and prevalence of certain bacterial species.
Han et al., 2017 [45] China	Pre/post-intervention	AAP 1999 definition for generalized Aggressive Periodontitis	Rapid attachment loss and bone destruction, and possible familial aggregation of disease.	28 ± 1.41	2		SRP	No treatment	Subgingival plaque Sterile Gracey curettes	V4 region	1 month	There was a lower Shannon index and higher Chao1 index post-treatment. Bacteroidetes, Spirochetes, and Fusobacteria were related to pathogenicity while Actinobacteria and Proteobacteria were associated with resolution of clinical symptoms.
Belstrom et al., 2018 [38] Denmark	Pre/post-intervention	AAP 2015 definition for moderate/severe chronic periodontitis	PD ≥ 5 & <7 mm and CAL 3–5 mm/ PD ≥ 7 mm and CAL ≥ 5 mm	47–75	25		SRP	Before SRP	Subgingival plaque Sterile Gracey curette	V3–V4 region	12 weeks	After SRP, there was a decrease in the relative abundance of periodontitis-associated genera in the subgingival plaque. There was a decrease in alpha diversity significantly after 2 and 6 weeks.
Chen et al., 2018 [42] USA	Pre/post-intervention	Chronic periodontitis	NA	NA	19		SRP	Before SRP	Subgingival plaque Paper point	V4 region	4 weeks	SRP was effective in decreasing the relative abundances of periodontitis-associated bacteria.
**Antibiotics used as adjuncts to SRP**												
Junemann et al., 2012 [48] Germany	Double-blind, parallel-group, placebo-controlled RCT	Generalized severe chronic periodontitis	More than 38% of sites with pocket probing depths of 6 mm or more	N/A	2	2	SRP + 500 mg amoxicillin + 400 mg metronidazole, three times daily × 7 days	SRP	Subgingival plaque Paper point	V6 region	2 months	For both the intervention group, there was an increase in alpha diversity. There was a shift in bacterial composition from Gram-negative Bacteroidetes to Gram-positive bacteria.
Laksmana et al., 2012 [50] USA	Pre/post-intervention	AAP definition of Aggressive Periodontitis	Rapid attachment loss and bone destruction, and possible familial aggregation of disease.	N/A	2		SRP + 500 mg amoxicillin + 500 mg metronidazole, three times daily × 8 days	N/A	Subgingival plaque Paper point	V4 region	8 weeks	The subgingival microbiome changed after therapy. After an intervention, there was an increase in the Gram-positive and Gram-negative commensals while a decrease in the bacteria of the Red complex.
Bizzaro et al., 2016 * [39] Netherland	Single blinded RCT	Chronic periodontitis	Proximal attachment loss of ≥3 mm at ≥2 non-adjacent teeth + below-median responders	NA	9	10	SRP+ 0.12% CHX rinse (2 × day × 28 days) and antibiotics (amoxicillin 375 mg + metronidazole 250 mg, 3 times a day × 7 days)	SRP+ CHX 0.12% only	Subgingival plaque Paper point	V5–V7 region	12 months	Antibiotic usage after 3 months resulted in significant changes in the subgingival microbiome. SRP+CHX predicted better clinical outcomes.
Hagenfeld et al., 2018 * [43] Germany	Double-blind, parallel-group RCT	AAP 1999 definition for moderate/severe chronic periodontitis	3 mm to <5 mm/_5 mm attachment loss	NA	42	47	SRP + 50 mg amoxicillin and 400 mg metronidazole	SRP + placebo	Subgingival plaque Paper points	V3–V4 region	8 weeks	Adjunctive use of antibiotics with SRP significantly reduced the richness, decreased the periodontitis associated genera, and changed the bacterial compositional structure.
de Oliveira et al., 2021 * [41] Brazil	Double-blind, placebo- controlled, RCT with two parallel arms	Untreated periodontitis	≥1 site with PD ≥ 6 mm and sites with PD ≥ 5 mm in different teeth		24	24	Subgingival instrumentation and probiotics	Subgingival instrumentation only	Subgingival plaque Sterile Gracey curettes	N/A	1 month	Short-term use of systemic probiotics with subgingival instrumentation did not show any additional clinical or microbiological improvement for the treatment of periodontitis.
Lu et al., 2021 * [51] China	RCT	Severe periodontitis: stage III/IV, grade B/C generalized periodontitis	At least six non-adjacent sites of six teeth with PD ≥ 5 mm and more than 30% of sites with radiographic bone loss > 1/2 of the root	T: 43.57 ± 6.63 C: 42.57 ± 3.29	7	7	SRP+ Amoxycillin (550 mg) and metronidazole (200 mg) three times a day for 7 days	SRP only	Subgingival plaque Sterile Gracey curettes	V3 and V4 region	6 months	The test group showed greater improvement in periodontal health than the control group. The test group showed lower microbial richness and diversity and less abundant *Porphyromonas*.
**Chemical agents**												
Teng et al., 2016 * [18] China	A double-blind randomized controlled trial	Experimental gingivitis	21 days gingivitis model	18–53	41	50	CPC mouth rinse, two times daily	Water rinse	Supragingival Sterile Gracey curette	V1–V3 region	3 weeks	The α and β diversity revealed that CPC treatment prevents the acquisition of new taxa that would otherwise accumulate but maintains the original biodiversity of healthy plaques.
Califf et al., 2017 [40] USA	Clinical trial	Periodontitis	At least four separate teeth with a pocket depth of 6 mm	41	17	17	0.25% sodium hypochlorite	Water rinse	Subgingival plaque Sterile Gracey curette	V4–V5 and shotgun was done as well.	3 months	The high diversity and number of metabolites were both significantly related to periodontal deep pockets. For the effectiveness of treatment metabolic dynamisms were more indicative than shifts in composition in the community.
Al-Kamel et al., 2019 * [13] (Prevention study Yemen) (Split into two parts)	Triple blinded, placebo-controlled, parallel-arm RCT	Experimental gingivitis	21 days gingivitis model	22.22 ± 1.73	10	10	T1:1.25% NAC mouthwash T2: 0.2% CHX mouthwash	Placebo mouthwash	Subgingival plaque Paper point	V1–V3 region	3 weeks	CHX was more effective in preventing and reversing experimental gingivitis than NAC. There was a non-significant increase in the species richness and alpha diversity compared to baseline for placebo and NAC group while CHX resulted in a decrease in these parameters.
Al-Kamel et al., 2019 * [13] (Treatment study) Yemen					10	10	1.25% N-acetyl cysteine mouthwash	0.2% CHX mouthwash	Subgingival plaque Paper point	V1–V3 region	3 weeks	CHX use was associated with a significant decrease in species richness and alpha diversity while NAC caused no change.
Wang et al., 2021 [56] China	Split mouth randomized controlled clinical trial	Generalized chronic periodontitis	CAL loss in >30% of sites	28–57	20		SRP+ (−)-Epigallocatechin Gallate (EGCG) solution (delivered through scaler tip)	SRP+coolant	Subgingival plaque Paper point	V3–V4 region	6 months	SRP+EGCG improved the clinical parameters and the relative abundance of Tannerella forsythia compared to SRP alone.
**Toothpaste**												
Hong et al., 2020 [47] Korea	Double-blind placebo controlled RCT	Gingivitis or incipient periodontitis	N/A	T: 37.08 ± 11.08; C: 33.72 ± 11.74	40	40	Toothpaste containing pyrophosphate	Toothpaste without pyrophosphate	Supragingival plaque/calculus Swabs	V4–V5 region	12 weeks	The toothpaste containing pyrophosphate inhibited the dysbiosis of oral microbiome and prevented proliferation of pathogens (Haemophilus, Fusobacterium and Capnocytophaga) and showed significant differences in the α diversity from baseline to 12 weeks.
Huang et al., 2016 * [17] China	Double-blind RCT	Moderate gingivitis	At least 10 bleeding sites; mean MGI is from 1.0–2.5	33.80 ± 7.86	47	44	The brush-plus-rinse group: manual toothbrush and a toothpaste containing 0.321% sodium fluoride and 1.16% stannous chloride and 20 mL rinse with 0.0747% CPC for 30 s after brushing.	Brush twice daily with a manual brush and a 0.243% sodium fluoride toothpaste	Supragingival plaque Sterile Gracey curette	V1–V3 region	27 days	The brush-plus-rinse group exhibited lower α diversity than the brush-alone group and overall lower detection in taxa after treatment while the β diversity of brush alone group post-treatment showed resemblance to baseline.
Hagenfeld et al., 2019 * [44] Germany	Double-blind, two center RCT	Mild to moderate periodontitis	PPD of ≥4 mm in at least four teeth except for third molars	54.22	20	21	Toothpaste containing zinc substituted carbonated hydroxyapatite	Toothpaste with amine fluoride/stannous fluoride	Subgingival plaque Paper point	V4–V6 region	12 weeks	The toothpaste containing anti-adhesive HA did not induce any changes in the microbial composition compared to anti-adhesive and antimicrobial AmF/SnF_2._ There were no changes in the alpha diversity.
**Air polishing**												
Lu et al., 2019 * [52] China	Clinical trial	Periodontitis patients on maintenance therapy	Maintained subjects that had a stable condition with more than 20 teeth, bop (%) ≤ 25%, PPD ≤ 5 mm	50.18 ± 12.08	17	17	Air polishing	Ultrasonic scaling	Subgingival plaque Sterile Gracey curettes	V3–V4 region	12 weeks	There was a reduction in the microbial diversity, proportion of periodontitis-associated bacteria, and pathogenic metabolism after the use of ultrasonic (US) and air polishing (AP). Chao1 was significantly reduced at 2 weeks in both US and AP groups but increased significantly from weeks 2 to 8 to week 12. Similarly, the Shannon index decreased after treatment and then increased at week 12.
Kruse et al., 2020 * [49] Germany	Single-blinded, randomized controlled split-mouth study	Chronic periodontitis patient undergoing maintenance therapy	2 single-rooted with PPD = 5 mm and positive BOP or >5 mm with or without positive BOP	61.4 ± 10.6	10		Air polishing using trehalose powder	Ultrasonic scaling	Subgingival plaque Paper point	NA	3 months	Air polishing and sonic treatment have a similar effect on the subgingival microbiome.
**Other**												
Queiroz et al., 2017 [54] Brazil	Single blinded RCT	Chronic periodontitis with Class II furcation on molars	Horizontal furcation PD ≥ 4 mm	53.14	13	13	Beta-tricalcium phosphate/hydroxyapatite graft/ EMD+BONE	EMD only	Subgingival plaque Paper point	NA	6 months	Treatment with EMD alters the dysbiotic subgingival microbiome and decreases the pathogen richness and increases commensal abundance.
Woelber et al., 2019 [57] Germany	Single blinded RCT	Gingivitis	Mean GI index ≥ 0.5	27.2 (4.7)	15	15	An anti-inflammatory diet low in processed carbohydrates and animal proteins and rich in omega-3 fatty acids, vitamin c, vitamin d, antioxidants, plant nitrates, and fiber.	Western diet	Subgingival plaque Sterile Gracey curettes	V3–V4 region	6 weeks	There was no significant difference seen in the subgingival microbiome with both diets. The alpha diversity was slightly higher in the experimental group.

* Included in meta-analysis; RCT: Randomized clinical trial; SRP: Scaling and root planing; PPD: periodontal probing depth; CAL: clinical attachment level; BOP: Bleeding on probing; NAC: N-acetyl cysteine CHX: Chlorhexidine; GI: Gingival index; MGI: Mazza Gingival index; AAP: American Association of Periodontology; CPC: Cetylpyridinium Chloride; EMD: emodogain.

**Table 2:** Description of microbial data analysis from the included studies.

Author and Year	Data Analysis	Statistical/Computational Methods	Alpha Diversity	Beta Diversity
Lu et al., 2021 [51]	QIIME2 & DADA2 pipelines were used for the generation of ASVs and α (Chao1, Shannon index, and richness) and β (PCoA) diversity were assessed. Taxonomies were assigned using HOMD	Pre- and post-treatment compared using paired *t*-test or Wilcoxon signed-rank test. The correlation of microbiota was analyzed using the Spearman correlation coefficient and heatmaps were generated.	Decrease in the Chao1, Shannon index, and richness in the test group and an increase in the control group.	PCoA showed dissimilarities among the three-time points (baseline, 3 months, and 6 months) in the two groups.
de Oliveira et al., 2021 [41]	OTUs were classified using NeoRefDB database. Shannon index, Bray-Curtis dissimilarity index, and PCoA plot were used.	Baseline and post-treatment were assessed by the Wilcoxon and Mann-Whitney test. Categorical variables were analyzed using Chi-square, McNemar, or Fisher test.	A significant decrease in the α diversity was observed for both the groups but not between therapies.	N/A
Wang et al., 2021 [56]	The mean relative abundance was calculated for Red-complex pathogens and HOMD was used for comparisons.	Relative abundances in groups were assessed using the Wilcoxon signed-rank test.	N/A	N/A
Hong et al., 2020 [47]	QIIME 2 and DADA2 pipelines were used for ASVs generation. Taxonomy assigned using Greengenes database taxonomy via feature classifier classify-sklearn.	For comparing β diversity ANOSIM was used. To compare α diversity and taxonomies between groups Wilcoxon’s rank-sum test or *t*-test was conducted. LDA and effect size (LEfSe) analysis was done. Linear regression analysis of specific strains was performed.	The α diversity was reduced for both the test and control groups at 12 weeks.	The PCoA plot showed a significant difference between baseline and 12 weeks.
Kruse et al., 2020 [49]	For bacteria identification, BLAST program was used for identifying bacterial sequences of 4 bactrial isolates (*Prevotella tannerae*, *Anaeroglobus geminatus*, *Actinomyces* sp. *oral taxon*, *Filifactor alocis*).	Paired *t*-test was used for temporal changes in bacterial concentration from baseline to post-treatment.	N/A	N/A
Al-Kamel et al., 2019 [13]	The MOTHUR (UCHIME) pipeline was used. Taxonomies assigned using SILVA, Greengenes database, Wang’s Bayesian classifier. BLASTN-based taxonomy assignment was used for classifying species levels. The HOMD, HOMD-ext, modified Greengene Gold set, and NCBI’s microbial 16S set were used. USEARCH was used for out cut-off. QIIME was used for the calculation of species richness, coverage, and α and β diversity indices. Subject clustering was done with PCoA based on the Jaccard distance metric.	Within-group differences were observed using Wilcoxon signed-rank test and with other groups using Mann–Whitney U test. LDA and effect size (LEfSe) analysis was conducted.	In the treatment sub study, there was a decrease in the richness and α diversity	The PCoA plots showed variations in the microbiome among the samples. Three major clusters were formed
Hagenfeld et al., 2019 [44]	DADA2 and Phyloseq R packages were used for the analysis of α diversity measurement, richness, and the number of observed RSV. The β diversity was measured using the Bray-Curtis distance matrix.	ANCOVA and likelihood ratio test was used.	There were no changes in the α diversity between the two groups.	The PCoA did not change in the test and control groups.
Lu et al., 2019 [52]	QIIME was used and OTU was clustered and compared against the HOMD. The α & β diversity was assessed. PCoA was performed using the Bray-Curtis distance.	Wilcoxon signed-rank test was used for comparing α diversity. Microbial differences in pockets were compared with the Mann–Whitney test. Heatmap was generated genera distribution and Spearman correlation was performed for co-occurrence networks.	There was richness, and the Shannon diversity was reduced after treatment with ultrasonic scaling and air polishing showed increased richness.	The PCoA plot revealed a close distribution of microbial communities among the ultrasonic group and air polishing group
Woelber et al., 2019 [57]	MOTHUR was used for sequence processing and compared with the SILVA 16S database. The R packages phyloseq and vegan packages were used for microbiome data analysis. The α diversity and richness were assessed.	Kruskal-Wallis test was conducted for comparisons.	The α diversity and richness showed a reduction over time and was overall higher for the experimental group.	The PCA plots showed that the composition of the subgingival microbiome varied between different patients and between the two sampling times of the same patient.
Hagenfeld et al., 2018 [43]	The DADA2, DECIPHER, and Phyloseq R packages were used. RSVs were classified according to the SILVA database. The richness, Shannon index, and Pielou index were measured for α diversity, and for β diversity, the Bray-Curtis distance and PCoA plots were measured. The differential abundance RSV was analyzed using DESeq2.	Mann–Whitney U test was used to compare both treatment groups and Wilcoxon signed-rank test was used before and after for each treatment group.	There was no difference in the richness, evenness, and α diversity within the control group, but richness decreased in the treatment group.	Bray-Curtis dissimilarity increased in both groups In the PCoA plot, the antibiotic group showed a clear separation of microbiomes before and after treatment and the placebo group showed no difference.
Chen et al., 2018 [42]	The OTU was generated, and the sequences were annotated by the RDP naïve Bayesian 16S classifier. STAMP was used for differential abundance and community analysis. The vegan package and metagenome package in R was used for α and β diversity.	N/A	The α diversity was similar among the test and the control group.	PCoA showed a clear separation of treatment and control groups.
Belstom et al., 2018 [38]	BLAST program was used for taxonomic assignment and compared with the HOMD. The relative abundance was calculated as the percentage of DNA reads assigned to each reference.	Relative abundances were compared using the Kruskal-Wallis and Mann-Whitney test with Benjamini Hochberg analyses. Spearman signed-rank test was used to compute the correlation of relative abundance in the samples	The αdiversity decreased after treatment.	N/A
Queiroz et al., 2017 [54]	The OTU was assigned by alignment to HOMD using the BLASTn algorithm. QIIME was used for microbial core analysis.	Parametric tests.	The treatment groups resulted in an increase in health-associated bacteria.	N/A
Han et al., 2017 [45]	QIIME was used for analysis. OTU were picked and clustered using UPARSE pipeline and RDP classifier and Greengenes database was used for assigning taxonomies. MUSCLE software was used for getting phylogenetic relationships. The Shannon index, Choa1 index, Simpson index, and richness was calculated. Rarefaction curves were generated. The UniFrac PCoA plots and UPGMA analysis were done.	N/A	The α diversity decreased while the richness increased after treatment	PCoA showed that pre-interventional and post-interventional samples were different.
Liu et al., 2018 [14]	QIIME and MOTHUR were used for data analysis. OTU was formed by USEARCH, and RDP was used for classifying into taxonomic groups based on the HOMD. PCA and PCoA plots were generated.	The α diversity and the differences in the pre- and post-treatment were compared using the Mann-Whitney U test. ANOSIM was used to compare the intra and inter-group similarities. ANCOVA was used to calculate the differences in the relative abundances. Spearman correlation was used for correlations between OTUs. LDA and effect size (LEfSe) analysis was conducted.	No difference in the Shannon index.	PCoA showed there was a difference in the bacterial composition before and after treatment.
Califf et al., 2017 [40]	The OTU counts were picked with UCLUST against the Greengenes database. Faith’s phylogenetic diversity and Choa1 index were assessed. QIIME was used to analyze β diversity the UniFrac distance, PCoA, and Bray-Curtis distance. Shotgun sequencing was done, and human reads were removed with KneadData. The bacterial reads were analyzed using the HUMAnN2 and MetaPhlAn2 pipeline.	Spearman rank correlation was used for correlation and the Mantel test was used.	Higher diversity correlated with periodontal pocket depth.	PCoA showed there was no compositional separation of microbiomes among different groups of disease severity.
Bizzarro et al., 2016 [39]	The OTU was randomly subsampled. The data analyses consisted of the Shannon diversity index, PCA, PERMANOVA using the Bray-Curtis similarity measure, and the Bray-Curtis similarity matrix.	Mann–Whitney test was used for assessing differences between groups at genus level and Wilcoxon signed-rank test was used for assessing the effects of time. ANOVA and ANCOVA were used for changes in Shannon diversity Pearson and Spearman were measures of correlation and Bray-Curtis and Kullback-Leibler were used for dissimilarity	The α diversity was similar between the test and control groups and similar within each group.	A significant difference between the treatment and control groups and within a group.
Huang et al., 2016 [17]	MOTHUR package was used and assigned sequences were classified using oral CORE reference database. The vegan R package was used for α and β diversity. PCoA and JSD matrix were constructed.	ANCOVA was used for treatment group comparisons.	There was a decrease in the alpha diversity in the treatment group	PCoA showed variation in the treatment group associated with gingival health.
Teng et al., 2016 [18]	MOTHUR package for analysis of Shannon index, richness and PCA.	To compare within subjects, the Wilcoxon rank-sum test was used and to compare two groups. Wilcoxon signed-rank test was used and for correlations, Spearman correlation analysis was carried out. FDR corrections were performed.	The α diversity in the treatment group remained stable while it increased in the control group.	The PCA plots showed there was structural segregation of the microbial community between the two groups after treatment
Shi et al., 2015 [23]	16S rRNA sequences were extracted from the shotgun sequencing data and aligned against the SILVA rRNA database, HOMD, and OSU CORE database. The vegan R package was used for rarefaction analysis of sequencing depth. QIIME was used for estimating the Shannon index, weighted UniFrac, & PCoA.	ANOSIM was conducted using MOTHUR for assessing microbiome similarities. Paired-t-test was used in all analyses. Hierarchical clustering was performed on the relative abundance profiles. The Spearman rank correlation was done for the distance measurement. Heat map was generated.	Alpha diversity decreased after the intervention.	PCoA showed a difference in the microbial composition at baseline and post-intervention
Schwarzberg et al., 2014 [55]	QIIME was used for data analysis and OTU was clustered using the UCLUST protocol using the Greengenes reference sequence. RDP classifier was used for taxonomic assignment. The relative abundance *Streptococcus*, *Prevotella* and *Fusobacterium* was assessed. The UniFrac based PCoA plots were constructed.	N/A	Increased relative abundance of *Fusobacterium* was correlated with pocket depths in samples. Individual subjects had different flora after intervention.	No difference in PCoA plot was observed for before and after treatment.
Laksmana et al., 2012 [50]	Libcompare and Pyrosequencing pipelines were used from RDP. Results were then compared to HOMD.	The % composition and cumulative % of total reads of species/phylotypes were observed.	N/A	N/A
Junemann et al., 2012 [48]	OTU clustering, rarefaction curves, and species richness estimator ACE were assessed using ESPIRIT. GAST pipeline and SILVA rRNA database was used for taxonomic annotation. Shannon and Simpson diversity indices were computed using the vegan R-package.	N/A	Alpha diversity (Shannon index and Simpson index) increased after intervention	N/A
Yamanaka et al., 2012 [58]	OTU clustered using UCLUST, & Greengenes database were used. Unifrac calculated by FastUnifrac. OTU, Chao1 index, and ACE index was calculated using the Vegan package in R.	Paired t-test and student t-test were performed to compare the matrices pre-and post-therapy and between individuals. Wilcoxon signed-rank test to compare relative abundances.	After the intervention, there was a reduction in Chao1 and Shannon index.	A significant compositional change was seen after intervention and showed strong distinct clustering.

ANCOVA: Analysis of covariates; ANOSIM: analysis of similarity; ANOVA: Analysis of variance; ASV: Actual sequence variant; JSD: Jensen Shannon divergence (JSD) matrix; HOMD: Human Oral Microbiome Database; LDA: Linear discriminant analysis; PCoA: Principal Coordinate Analysis; PCA: Principal component analysis; PERMANOVA: one-way permutational multivariate analysis of variance; RDP: Ribosomal Database Project; RSV: ribosomal sequence variants; UPGMA: Unweighted Pair Group Method with Arithmetic mean.

## Data Availability

All data generated or analyzed during this study will be included in a published article (and its supplementary information file).

## Supplementary Materials

The following are available online at https://www.mdpi.com/article/10.3390/microorganisms10081582/s1, Figure S1: Meta-analysis of richness post-treatment (treatment vs. control); Figure S2. Meta-analysis of Shannon index post-treatment (treatment vs. control); Figure S3. Meta-analysis of Chao1 index post-treatment (treatment vs. control); Figure S4. Galbraith scatter plot of richness; Figure S5. Galbraith scatter plot of Chao1 index; Figure S6. Galbraith scatter plot of Shannon index; Figure S7. Leave one out analysis richness; Figure S8. Leave one out analysis Shannon index; Figure S9. Leave one out analysis Chao1 index; Figure S10. Funnel plot richness; Figure S11. Funnel plot Shannon index; Figure S12. Funnel plot Chao1 index; Figure S13. Risk of bias assessment; Figure S14. GRADE assessment.

## Appendix A

**Table A1:** Search strategy.

Database	Search String	Results
**Medline(PubMed)**	((((“Metagenomics” [MeSH Terms] OR “Metagenome” [MeSH Terms] OR “High-Throughput Nucleotide Sequencing” [MeSH Terms] OR “microbiota” [MeSH Terms] OR “genes, bacterial” [MeSH Terms] OR “Metagenomics” [Title/Abstract] OR “16S rDNA” [Title/Abstract] OR “16S rRNA” [Title/Abstract]) AND (“Or” [All Fields] AND “Pyrosequencing” [Title/Abstract])) OR “next-generation sequencing” [Title/Abstract] OR “Illumina sequencing” [Title/Abstract] OR “Functional gene array” [Title/Abstract] OR “Oral microbiome” [Title/Abstract] OR “Bacterial diversity” [Title/Abstract] OR “Bacterial community” [Title/Abstract]) AND (“Tooth Diseases” [MeSH Terms] OR “Mouth Diseases” [MeSH Terms] OR “Gingival diseases” [MeSH Terms] OR “Gingivitis” [MeSH Terms] OR “Periodontal diseases” [MeSH Terms] OR “Periodontal debridement” [MeSH Terms] OR “Periodontal index” [MeSH Terms] OR “Periodontal pocket” [MeSH Terms] OR “probing depth” [Title/Abstract] OR “periodont*” [All Fields] OR “plaque score” [All Fields]) AND “humans” [MeSH Terms]) AND (humans [Filter])	1020
**Scopus**	(TITLE-ABS-KEY (“Metagenomics” OR “Metagenome” OR “High-Throughput Nucleotide Sequencing” OR “microbiota” OR “Genes, Bacterial” OR “metagenomics” OR “16S rDNA” OR “16S rRNA” OR pyrosequencing OR “next-generation sequencing” OR “Illumina sequencing” OR “Functional gene array” OR “Oral microbiome” OR “Bacterial diversity” OR “Bacterial community”) AND TITLE-ABS-KEY (“Tooth Diseases” OR “Mouth Diseases” OR “Gingival diseases” OR “Gingivitis” OR “Periodontal diseases” OR “Periodontal debridement” OR “Periodontal index” OR “Periodontal pocket” OR “probing depth” OR “periodont*” OR “plaque score”)) AND (LIMIT-TO (EXACTKEYWORD, “Human”)) AND (EXCLUDE (DOCTYPE, “re”) OR EXCLUDE (DOCTYPE, “ed”) OR EXCLUDE (DOCTYPE, “le”) OR EXCLUDE (DOCTYPE, “no”) OR EXCLUDE (DOCTYPE, “cp”) OR EXCLUDE (DOCTYPE, “ch”) OR EXCLUDE (DOCTYPE, “sh”) OR EXCLUDE (DOCTYPE, “tb”) OR EXCLUDE (DOCTYPE, “Undefined”))	2274
**Dentistry & Oral Sciences source**	(“Metagenomics” OR “Metagenome” OR “High-Throughput Nucleotide Sequencing” OR “microbiota” OR “Genes, Bacterial” OR “metagenomics” OR “16S rDNA” OR “16S rRNA” OR pyrosequencing OR “next-generation sequencing” OR “Illumina sequencing” OR “Functional gene array” OR “Oral microbiome” OR “Bacterial diversity” OR “Bacterial community”) AND (“Tooth Diseases” OR “Mouth Diseases” OR “Gingival diseases” OR “Gingivitis” OR “Periodontal diseases” OR “Periodontal debridement” OR “Periodontal index” OR “Periodontal pocket” OR “probing depth” OR “periodont*” OR “plaque score”)	1444
**ProQuest Dissertation**	“Oral microbiome” OR “16S rDNA” OR “16S rRNA” AND (“Gingival diseases” OR “Gingivitis” OR “Periodontal diseases”) AND “clinical trial” AND filter (dissertations)	25
**OpenGrey database**	“Oral microbiome” OR “16S rDNA” OR “16S rRNA”AND (“Gingival diseases” OR “Gingivitis” OR “Periodontal diseases”) AND “clinical trial”	0
**Cochrane database of systematic reviews**	“Oral microbiome” OR “16S rDNA” OR “16S rRNA” AND (“Gingival diseases” OR “Gingivitis” OR “Periodontal diseases”) AND “clinical trial”	256

**Table A2:** Data extraction form.

Variable		Definition
**Study Characteristics**		
1.	SID	Unique identification number of study
2.	Author	Last name of first author
3.	Year	Year of publication
4.	Country	Country of study conducted
5.	Study design	Design of the study (e.g., parallel or split)
6.	Randomization	Describes if randomization was performed (Yes = 1, No = 2, Not clear = 99)
7.	Blinding	Describes if blinding was done and level of blinding (Single = 1, double = 2, triple = 3, none = 4, not clear = 99)
8.	Sample size	Did the researchers calculate the sample size (Yes = 1, No = 2, Not clear = 99)
**Participant Characteristics**		
9.	Cases_n	Total number of cases
10.	Control_n	Total number of control
11.	age_mean, age_sd	Age of included participants [mean (SD), median (IQR), or categorical age, as reported]
12.	Periodontal disease type	Describe the periodontal condition
13.	Periodontal definition	Describe the definition used for classifying periodontal disease
14.	Test description	Describes the intervention used for test group
15.	Control description	Describes the intervention/placebo/no treatment for the control group
16.	Treatment duration	Describes the recalls, and total duration of intervention
**Collection and extraction method**		
17.	Plaque_collection	Describes the site for plaque collection (supra or subgingival) and the instrument used for collection (e.g., paper point or sterile curette)
18.	Hypervariable region	Describes the variable region used for DNA extraction
**Measures of alpha diversity**		
19.	OTU count (richness)	The OTU count scores at baseline and post-intervention; mean and SD
20.	Chao1 index	The Chao1 scores at baseline and post-intervention; mean and SD
21.	Shannon diversity index	The Shannon diversity scores at baseline and post-intervention; mean and SD
**Outcome**		Overall results of the study.

**Table A3:** The relative abundances of bacteria.

Author and Year	Pre-Intervention Abundant Taxa	Post-Intervention Abundant Taxa	Post-Intervention Increase	Post-Intervention Decrease	Overall Abundant Taxa
Lu et al., 2021	*Porphyromonas*, *Treponema*, *Prevotella*, *Fusobacterium*, *Filifactor*, *Saccharibacteria TM7 G-5*, and *Peptostreptococcaceae XIG-6*,	*Actinomyces* and *Capnocytophaga*	*Actinomyces*, *Rothia*, *Neisseria*, *Capnocytophaga*, *Lautropia*, and *Cardiobacterium*	*Porphyromonas*, *Treponema*, *Filifactor*, *TM7 G-5*, *Peptostreptococcaceae XI G-6*, *Fretibacterium*, *Dialister*, and *Peptococcus*.	*Porphyromonas*, *Treponema*, *Prevotella*, *Fusobacterium*, *Filifactor*, *Saccharibacteria*, *Peptostreptococcaceae XIG-6*, *Actinomyces* and *Capnocytophaga*
de Oliveira et al., 2021	*Capnocytophaga* spp., *Enterobactereacea*, *P.acnes*, *Staphylococus CN.*	*Actinomyces* spp. *Aggregatibacter actinomycetemcomitans*, *Acinetobacter baumannii*, *Clostridium difficile*, *Candida albicans*, *Campylobacter* spp., *Corynebacterium matruchotii*, *Dialister pneumosintes*, *Eikenella corrodens*, *Eubacterium* spp., *Enterococcus faecalis*, *Fusobacterium periodonticum*, *Fusobacterium nucleatum*, *Filifactor alocis*, *Gemella* spp., *Hafnia alvei*, *Helicobacter pylori*, *L.buccalis*, *Lactobacillus* spp. *Olsenella uli*, *Parvimonas micra*, *Paeriginosa*, *Porphyromonas gingivalis*, *Prevotella* spp., *streptococci* spp. *Staphylococcus aureus*, *Treponema socranskii*, *Treponema denticola*, *Tannerella forsythia*, *Veillonella parvula*	N/A	N/A	*Prevotella* spp., *Tannerella forsythia*, *Treponema denticola*, *Pseudomonas aeruginosa*, *Porphyromonas gingivalis*, *Actinomyces* spp.
Wang et al., 2021	Red complex bacteria	N/A	N/A	*Porphyromonas gingivalis* and *Treponema denticola*, *Tannerella* *forsythia*	*Porphyromonas gingivalis* and *Treponema denticola*, *Tannerella* *forsythia*
Hong et al., 2020	*Firmicutes*, *Proteobacteria*	*Streptococcus*	*Firmicutes*	Phylum: *Spirochaetes*, *Proteobacteria*, *Fusobacteria.* Genus: *Haemophilus*, *Fusobacterium* and *Capnocytophaga*	Phylum: *Firmicutes*, *Proteobacteria*, *Actinobacteria* Genus: *Streptococcus*, *Actinomyces*, *Lautropia*, *Actinomyces*, *Haemophilus*, *f_Neisseriaceae*, *Neisseria*, *Rothia*, *Corynebacterium*, *Capnocytophaga*
Kruse et al., 2020	Gram-positive aerobic cocci, Gram-positive aerobic rods, Gram-positive anaerobic rods, and Gram-negative anaerobic rods.	Gram-positive aerobic cocci, Gram-positive anaerobic cocci, Gram-positive anaerobic rods	*Slackia exigua*, *Eubacterium yurii*, *Atopobium rimae*, *Filifactor alocis*, *Bifidobacterium dentium*, *Solobacterium moorei*, *Olsenella uli*	*Actinomyces meyeri*, *Actinomyces oris*, *Actinomyces odontolyticus*, *Actinomyces naeslundii*, *Actinomyces gerencseriae*, *Corynebacterium matruchotii*, *Rothia mucilaginosa*, *Rothia aeria*, *Streptococcus oralis*, *Streptococcus mitis*, *Streptococcus sanguinis*, *Streptococcus cristatus*, *Streptococcus sinensis*, *Streptococcus salivarius*, *Streptococcus anginosus*, *Streptococcus constellatus*, *Streptococcus intermedius*, *Streptococcus mutans*, *Gemella morbillorum*, *Streptococcus* spp.	*Porphyromonas gingivalis*, *Prevotella intermedia*, *Prevotella nigrescens*, *Prevotella tannerae*, *Prevotella buccae*, *Fusobacterium nucleatum*, *Campylobacter rectus*, *Tannerella forsythia*, *Selenomonas* spp., *parvula*, *Dialister pneumosintes*, *Anaeroglobus geminaus*, *Anaeroglobus geminatus*, *Parvimonas micra*, *Capnocytophaga ochracea*, *Capnocytophaga gingivalis*, *Aggregatibacter aphrophilus*, *Cardiobacterium hominis*, *Eikenella corrodens*, *Neisseria macacae/mucosa*, *Neisseria elongate*, *Neisseria flavescens*, *Neisseria bacilliformis*, *Nesseria* spp., *Lautropia mirabilis*, *Actinomyces meyeri*, *Actinomyces oris*, *Actinomyces odontolyticus*, *Actinomyces naeslundii*, *Actinomyces gerencseriae*, *Corynebacterium matruchotii*, *Rothia mucilaginosa*, *Rothia aeria*, *Streptococcus oralis*, *Streptococcus mitis*, *Streptococcus sanguinis*, *Streptococcus cristatus*, *Streptococcus sinensis*, *Streptococcus salivarius*, *Streptococcus anginosus*, *Streptococcus constellatus*, *Streptococcus intermedius*, *Streptococcus mutans*, *Gemella morbillorum*, *Streptococcus*
Al-Kamel et al., 2019 Prevention sub-study	*Streptococcus*, *Fusobacterium*, *Leptotrichia*, *Veillonella*, *Haemophilus*, *Actinomyces*, *Lautropia*, *Rothia*, *Capnocytophaga*	NAC: *Fusobacterium*, *Streptococcus*, *Leptotrichia*, *Cardiobacterium*, *Campylobacter*, *TM7_G_1_.* CHX: *Fusobacterium. Streptococcus*, *Granulicatella*, *Neisseria*, *Capnocytophaga*.	NAC: *Haemophilus*, *Lautropia*, *Rothia*, *Kingella*, *Brevundimonas*, *Escherichia.* CHX: *Rothia*, *Actinomyces*, *Cardiobacterium*, *Porphyromonas*, *Peptostreptococcus*, *Actinobaculum*, *Lachnospiraceae_G_3_*, *Lachnoanaerobaculum.*	NAC: *SR1_G_1_*, *Selenomonas*, *Olsenella*, *Parvimonas*, *Dialister*, *Oribacterium*, *TM7_G_1_*, *Cardiobacterium*, *Campylobacter*, *Fusobacterium.* *CHX: Granulicatella*	*Fusobacterium*, *Streptococcus*, *Leptotrichia*, *Veillonella*, *Propionibacterium*, *Actinomyces*
Al-Kamel et al., 2019 Treatment sub-study	*Fusobacterium*, *Streptococcus*, *TM7_G_1_*, *Leptotrichia*, *Prevotella*, *Veillonella*, *Porphyromonas*	CHX: *Fusobacterium*, *Streptococcus*, *Capnocytophaga*, *Bacteroidales_G_2*, *Prevotella*, *Veillonella* NAC: *Fusobacterium*, *Streptococcus*, *TM7_G_1*, *Leptotrichia*, *Propionibacterium*, *Cardiobacterium*	*Capnocytophaga*	*Corynebacterium*, *Stomatobaculum*, *Selenomonas*, *SR1-G_1*, *Lachnospiraceae_G1*, *TM7_G_3_*, *Lachnoanaerobaculum*, *Gemella*, *Propionibacterium*, *Veillonella*, *Tannerella*, *Cardiobacterium*, *Actinomyces*, *TM7_G_1_*	*Fusobacterium*, *Streptococcus*, *TM7_G_1_*, *Leptotrichia*, *Prevotella*, *Veillonella*, *Propionibacterium.*
Hagenfeld et al., 2019	*Fusobacterium*, *Prevotella*, *Veillonella*	N/A	N/A	N/A	*Fusobacterium*, *Prevotella*, *Veillonella*, *Porphyromonas*, *Alloprevotella*, *Campylobacter*, *Treponema_2*, *Streptococcus*
Lu et al., 2019	*Streptococcus*, *and Saccharibacteria*	N/A	*Streptococcus*, *Escherichia*, *Acidopropionibacterium*, *Serratia*	*Treponema*, *Bacteroidacea*	*Actinomyces*, *Streptococcus*, *Leptotrichia*, *Capnocytophaga*, *Lautropia*, *Fusobacterium*, *Neisseria.*
Woelber et al., 2019	*Streptococcus*, *Veillonella*, *Fusobacterium*, *Actinomyces*, *Prevotella*	*Fusobacterium*, *Veillonella*, *Streptococcus*, *Actinomyces*, *Prevotella.*	*Prevotella*, *Veillonella*, *Rothia*	*Streptococcus*, *Fusobacterium*, *Campylobacter*, *Cornybacterium*	*Fusobacterium*, *Veillonella*, *Streptococcus*, *Actinomyces*, *Rothia*, *Prevotella.*
Hagenfeld et al., 2018	*Fusobacterium*, *Porphyromonas*, *Tannerella*, *Fretibacterium.*	N/A	*Streptococcus*, *Veillonella.*	*Porphyromonas*, *Tannerella*, *Treponema*, *Prevotella*, *Campylobacter*, *Fusobacterium*, *Parvimonas*, *Fretibacterium*, *Fillibactor*, *Oceanivirga.*	N/A
Chen et al., 2018	*Fillifactor*, *Desulfobulbus*, *Eubacterium*, *Hallella*, *Porphyromonas*, *Phocaeicola*, *Tanerella*, *Bacteroidetes*, *Alloprevotella*, *Johnsonella*, *Treponema*, *Leptotrichia*, *Mogibacterium*	N/A	N/A	N/A	*Fusobacterium*, *Prevotella*, *Porphyromonas*, *Treponema*, *Corynebacterium*, *Leptotrichia*, *Selenomonas*, *Actinomyces*, *Campylobacter*, *Tanerella (total 27 genus)*
Belstrom et al., 2018	*Prevotella*, *Treponema*, *Porphyromonas*, *Fusobacterium*	*Rothia*, *Prevotella*, *Streptococcus*	*Streptococcus*, *Rothia*, *Actinomyces*	*Porphyromonas*, *Treponema*	*Prevotella*, *Treponema*, *Porphyromonas*, *Fusobacterium*, *Rothia*, *Streptococcus*, *Cornyebacterium*, *Actinomyces.*
Quieiroz et al., 2017	*Fusobacterium*, *Pseudomonas*, *Streptococcus*, *Fillifactor*, *Parvimonas.*	N/A	N/A	*Selenomonas*, *Fillifactor*, *Fusobacterium*	*Actinomyces*, *Campylobacter*, *Fillifactor*, *Fusobacterium*, *Gemella*, *Parvimonas*, *Psedomonas*, *Propionibacterium*, *Selenomonas*, *Streptococcus*, *Haemophilus*, *Veillonella*
Han et al., 2017	*Sharpea*, *Moryella*, *Fusobacterium*, *Johnsonella*, *Peptostreptococcus*, *Peptococcus*, *Treponema*, *TG5*, *Desulfobulbus*, *Fillifactor*, *Tannerella*, *Porphyromonas*, *Megamonas*, *Esherichia*, *Selemonas*, *Dialister*, *Megasphaera*, *Prevotella*, *Leptotrichia*, *Hylemonella*, *Campylobacter*, *Bacteroides*, *Syntrophomonas*	*Kingella*, *Sphingopyxis*, *Lautropia*, *Capnocytophagam Neisseria*, *Aggregatibacter*, *Cornybacterium*, *Actinomyces*, *Parasscardovia*, *Veillonella*, *Rothia*, *Streptococcus*	*Actinobacteria*, *Proteobacteria*	*Bacteroidetes*, *Spirochaetes*, *Fusobacteria*	Phyla: *Bacteroidetes*, *Actinobacteria*, *Proteobacteria*, *Firmicutes*, *Fusobacteria*, *Spirochaetes*, *Synergistetes*
Liu et al., 2017	*Porphyromonas*, *Treponema*, *Fretibacterium*	*Streptococcus*, *Lautropia*, *Haemophilus*, *Actinomyces*	*Lautropia*, *Actinomyces*, *Haemophilus*	*Treponema*, *Porphyromonas*, *Fretibacterium*	*Neisseria*, *Streptococcus*, *Fusobacterium*
Califf et al., 2017	*Porphyromonas*, *Desulfovibrio*, *SHD-231*, *Treponema*, *Haemophilus*, *Acholeplasma*, *TG5*, *Mycoplasma*, *Eikenella*, *Desulfobulbus*, *Pseudoramibacter_Eubacterium*, *Methylobacterium* *Mogibacterium*, *Scardovia*	*Desulfovibrio*, *Streptococcus*, *Methanobrevibacter*, *PedobacterBE24*, *Butyrivibrio* *Peptococcus*, *Rhizobium*, *Aerococcus*, *Filifactor*, *Slackia*	N/A	N/A	*Desulfovibrio*, *Butyrivibrio*, *Methanobrevibacter*, *Pedobacter*, *Peptococcus*, *Filifactortreptococcus*, *Aerococcus*, *Slackia*
Bizarro et al., 2016	*Porphyromonas*, *Treponema*, *Fusobacterium*, *Fillifactor.*	*Actinomyces*, *Streptococcus*, *Veillonella*, *Neisseria*, *Haemophilus*	Both groups: *Neisseria*, *Rothia*, *Capnocytophaga*, *Streptococcus* Test group: *Veillonella*, *Haemophilus* Control group: *Parvimonas*, *Actinomyces*	Both groups: *Fillifactor*, *Tannerella*, *uncultured Clostridiales family xiii incertae sedis*, *Porphyromonas*, *Treponema*, *uncultured Synergistaceae* Test group: *Paludibacter*, *Fusobacterium*, *Parvimonas*	*Fusobacterium*, *Prevotella*, *Treponema*, *Parvimonas*, *Porphyromonas*, *Paludibacter*, *Neisseria*, *Rothia*, *Fillifactor*, *Actinomyces*, *Streptococcus*, *Veillonella*, *Tannerella*, *Uncult. Clostridiales*, *Capnocytophaga*, *Haemophilus*, *Campylobacter*
Teng et al., 2016	*Streptococcus*, *Actinomyces*, *Rothia*, *Veillonella*	*Leptotrichia*, *Neisseria*, *Capnocytophaga*, *Prevotella*, *Fusobacterium*, *Haemophilus*, *Lautropia*, *Abiotropia*	*Haemophilus*, *Lautropia*, *Neisseria*, *Capnocytophaga*, *Propioni-bacterium*	*Porphyromonas*, *Peptostreptococcus*, *Prevotella*, *Peptococcus*, *Selenomonas*, *Solobacterium*, *SR1*, *Tannerella*, TM7 genus, *Uncultured_Lachnospiraceae*, *Atopobium*, *Gemella*, *Megasphaera*, *Mogibacterium*, *Moraxella*, *Oribacterium* and *Shuttleworthia*	*Strptococcus*, *Leptotrichia*, *Actinomyces*, *Neisseria*, *Capnocytophaga*, *Rothia*, *Prevotella*, *Fusobacterium*, *Haemophilus*, *Lautropia*, *Porphyromonas*, *Cornyebacterium*, *Abiotropia*
Huang et al., 2016	*Prevotella*, *Leptotrichia*, *Selenomonas*, *uncultured Lachnospiraceae*, *TM7*, *Tannerella*, *Peptococcus*, *and unclassified Veillonellaceae*	*Rothia*, *Granulicatella*, *Bergeyella and Lautropia*	*Actinomyces*	*T-Actinobaculum*, *TM7 and Leptotrichia* *C-**Actinobaculum*, *TM7 and Leptotrichia*	*Rothia*, *Bergeyella*, *Lautropia*, *Granulicatella*, *Prevotella*, *Leptotrichia*, *Selenomonas*, *uncultured Lachnospiraceae*, *TM7*, *Tannerella*, *Peptococcus*, *and unclassified Veillonellaceae.*
Shi et al., 2015	*Porphyromonas*, *Treponema*, *Tannerella*, *Olsnella*, *Peptostreptococcus*, *Synergistes*, *Fillifactor*, *Mycoplasma*	*Actinomyces*, *Streptococcus*, *Rothia*, *Bergeyella*	*Actinomyces*, *Streptococcus*, *Rothia*, *Bergeyella.*	*Porphyromonas*, *Treponema*, *Tannerella*	*Prevotella*, *Fusobacterium*
Schwarzberg et al., 2014	N/A	N/A	*Streptococcus*, *Veillonella*	*Prevotella*, *Fusobacterium*, *Leptotrichia*	*Prevotella*, *Fusobacterium*, *Streptococcus*
Laksmana et al., 2012	*Fusobacterium*, *Porphyromonas*, *Prevotella*, *Synergistetes* spp., *Fillifactor*, *Actinomyces*, *Treponema*	*Fusobacterium*, *Porphyromonas*, *Prevotella*, *Streptococcus*, *Veillonella*	*Streptococcus*, *Rothia*, *Actinomyces*, *Veillonella*	*Fusobacterium*, *Porphyromonas*, *Treponema*, *Tannerella*	N/A
Junemann et al., 2012	*Porphyromonas*, *Prevotella*, *Treponema*, *Fusobacterium*, *Tannerella.*	*Prevotella*, *Streptococcus*, *Fusobacterium*	*Prevotella*, *Selenomonas*, *Streptococcus*, *Actinomyces*, *Rothia*	Test group: *Treponema*, *Fillifactor*, *Porphyromonas*, *Tannerella* Control group: *Porphyromonas*, *Tannerella*	*Phyla: Bacteroidetes*, *Firmicutes*, *Fusobacteria*, *Proteobacteria*, *Actinobacteria*, *Spirochaetes*, *and Synergistetes*
Yamanaka et al., 2012	*Streptococcus*, *Leptotrichia*, *Actinomyces*, *Rothia*, *Fusobacterium*	*Streptococcus*, *Leptotrichia*, *Actinomyces*, *Rothia*, *Corynebacterium*	*Corynebacterium*	*Fusobacterium*, *Kingella*	*Streptococcus*, *Prevotella*, *Veillonella*, *Rothia*, *Actinomyces*, *Neisseria*, *Porphyromonas*, *Gemelia*, *Fusobacterium*, *Leptotrichia*, *Granulicatella*, *Capnocytophaga*, *Corynebacterium*
