# Supplementary material for: Effect of Periodontal Interventions on Characteristics of the Periodontal Microbial Profile: A Systematic Review and Meta-Analysis
Source: Microorganisms. 2022 Aug 5;10(8):1582. doi: 10.3390/microorganisms10081582 (PMC9416518; doi:10.3390/microorganisms10081582)
Supplement: Supplementary file 1 [file microorganisms-10-01582-s001.zip › microorganisms-1819078-supplementary.pdf]

Supplementary figures:

Supplementary figure S1. Meta-analysis of richness post-treatment (treatment vs. control).

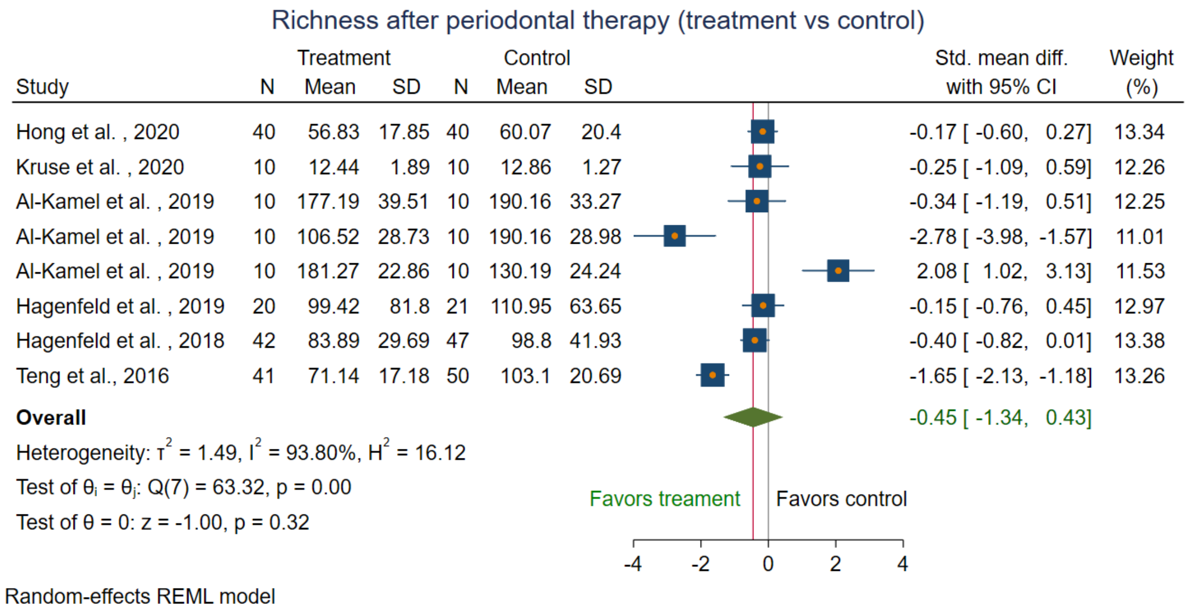

Supplementary figure S2. Meta-analysis of Shannon index post-treatment (treatment vs. control).

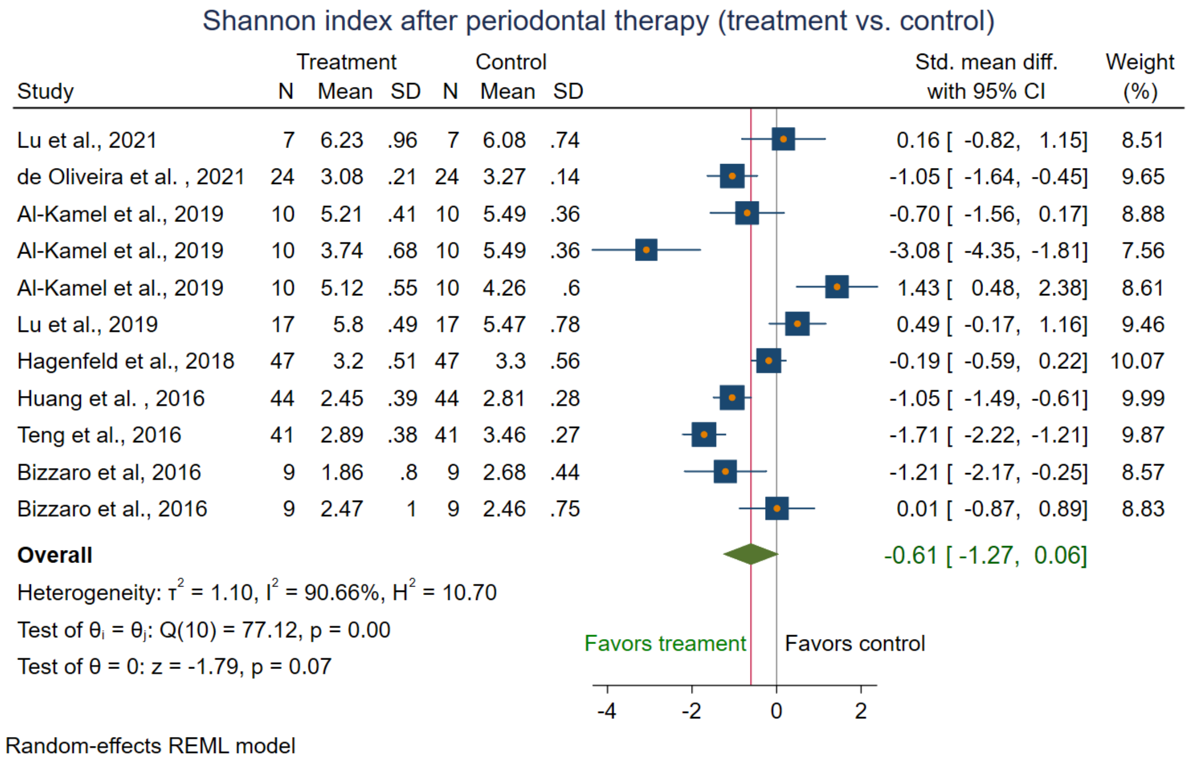

Supplementary figure S3. Meta-analysis of Chao1 index post-treatment (treatment vs. control).

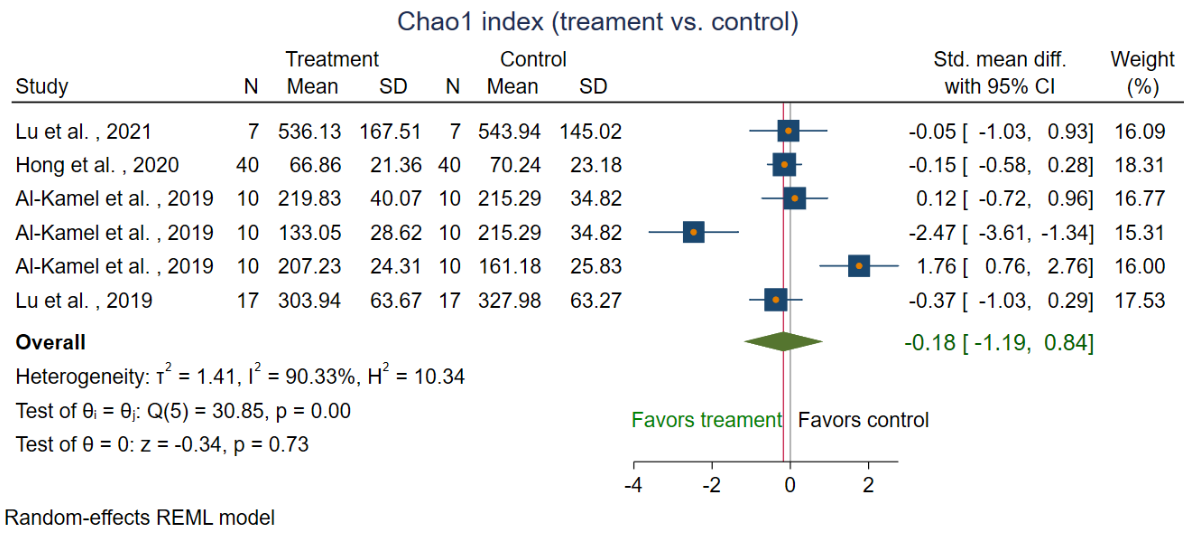

Supplementary figure S4. Galbraith scatter plot of richness

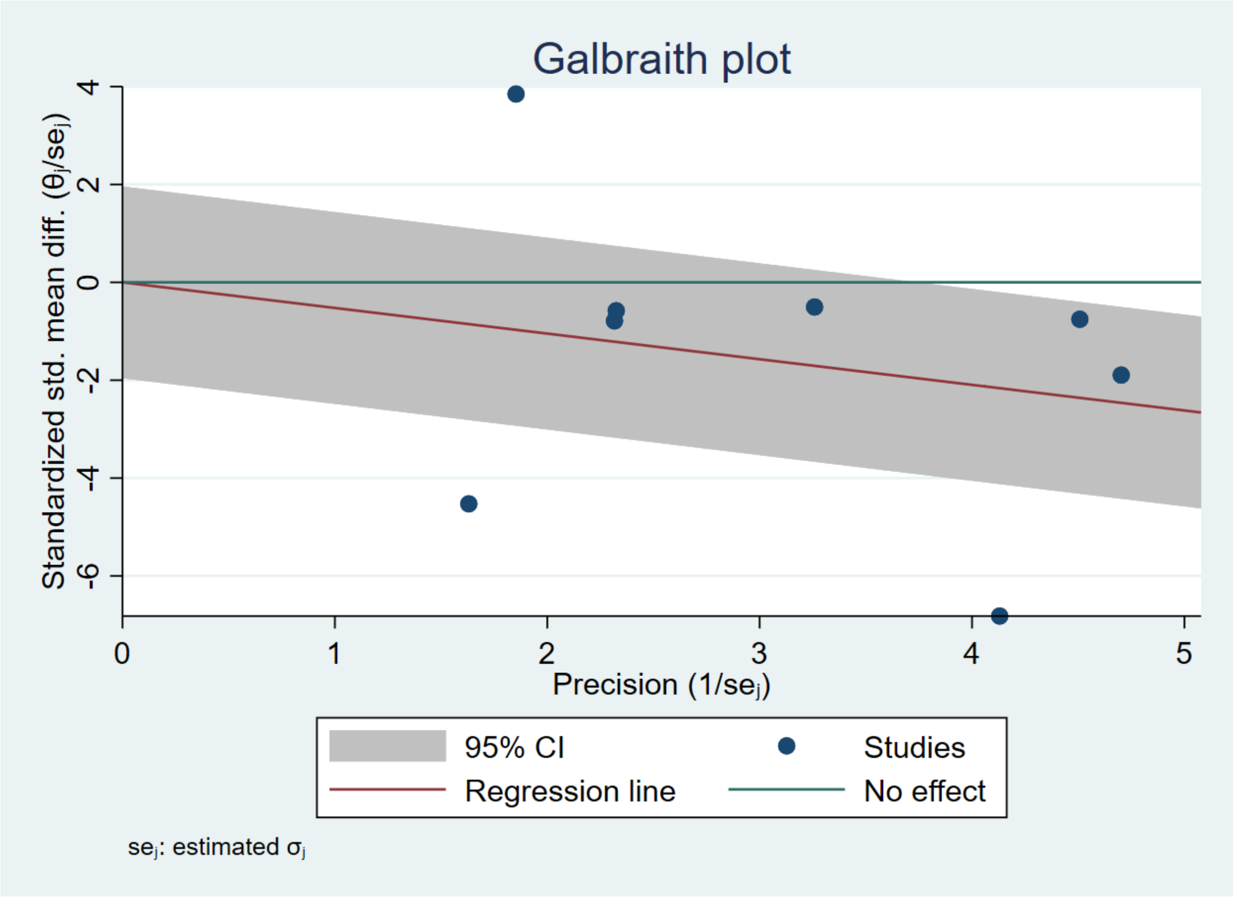

Supplementary figure S5. Galbraith scatter plot of Shannon index.

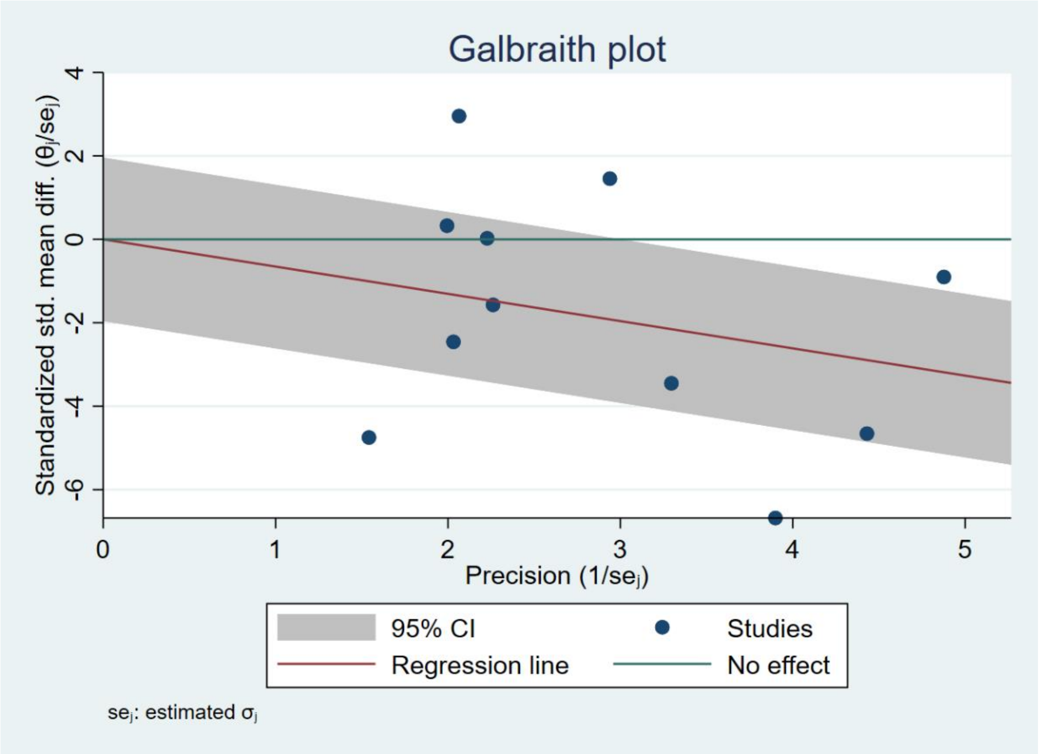

Supplementary figure S6. Galbraith scatter plot of Chao1 index.

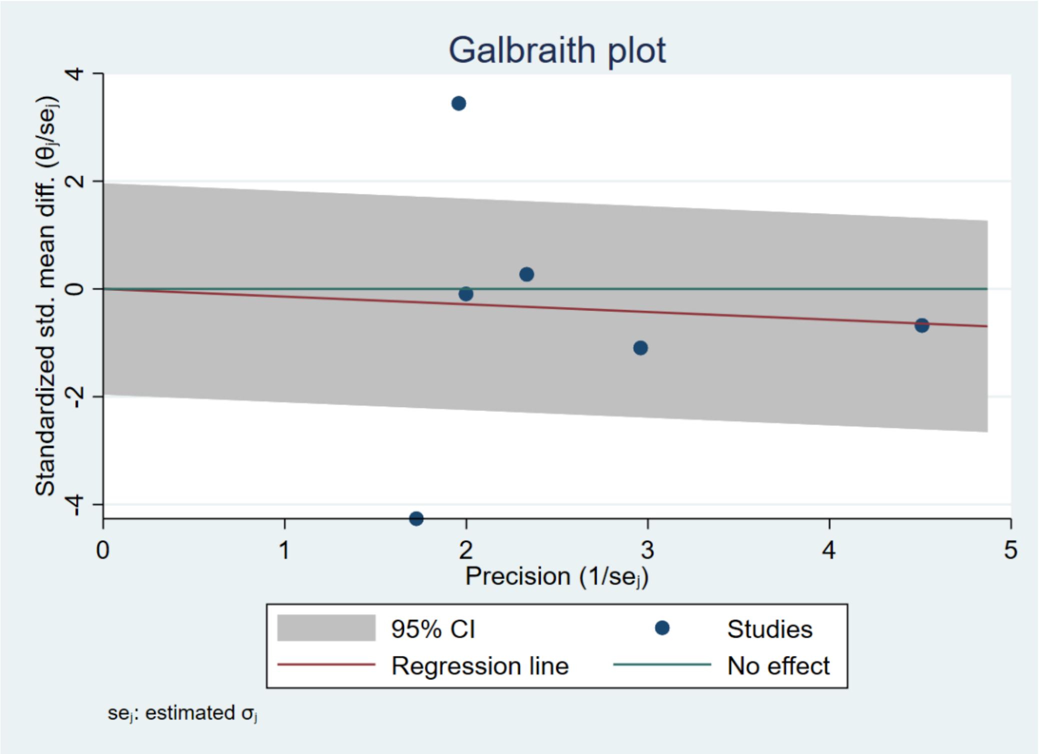

Supplementary figure S7. Leave one out analysis richness

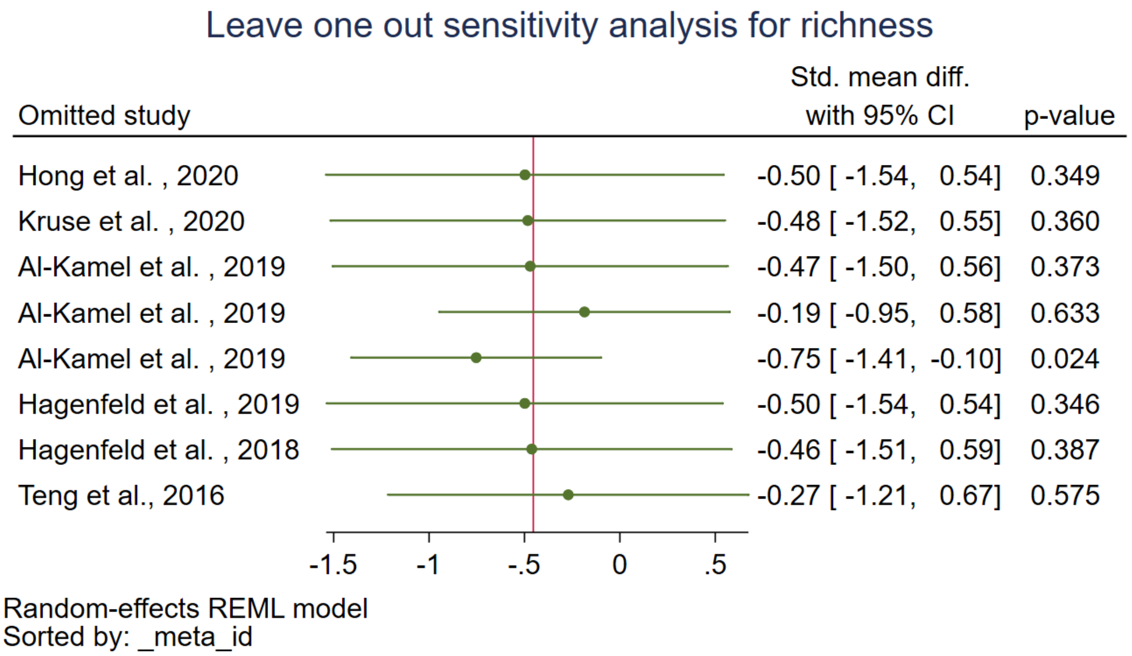

Supplementary figure S8. Leave one out analysis Shannon index.

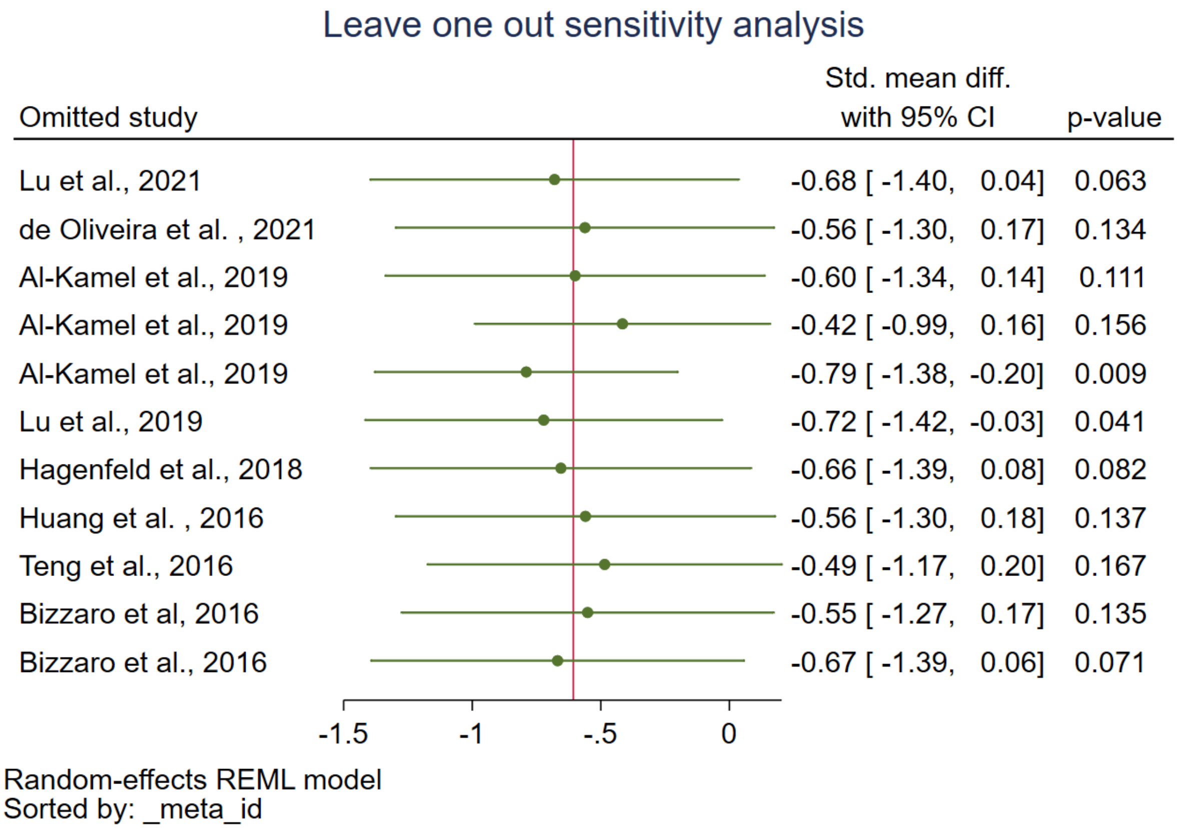

Supplementary figure S9. Leave one out analysis Chao1 index.

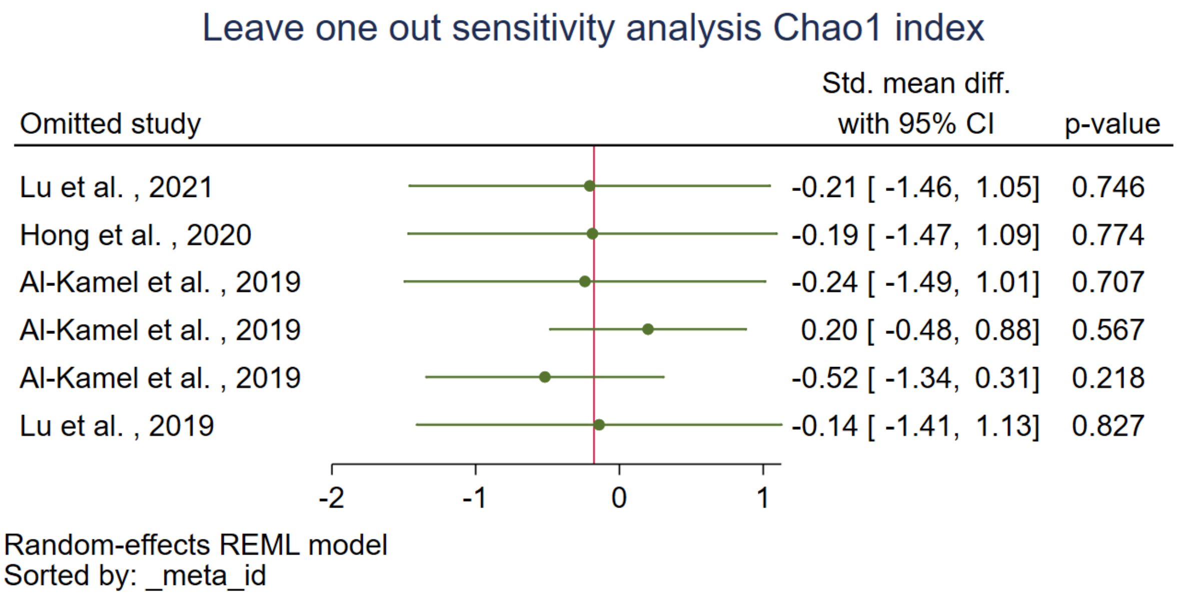

Supplementary figure S10. Funnel plot richness

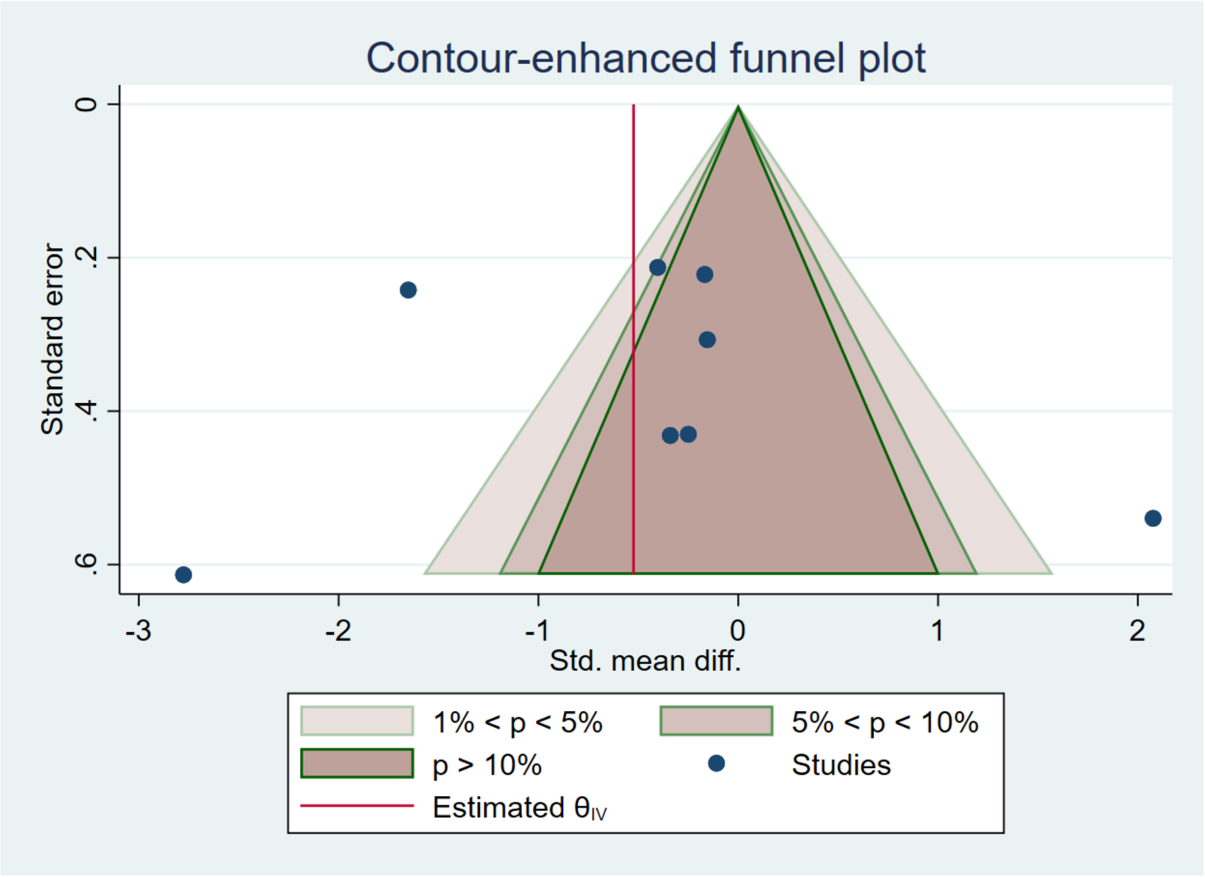

Supplementary figure S11. Funnel plot Shannon index

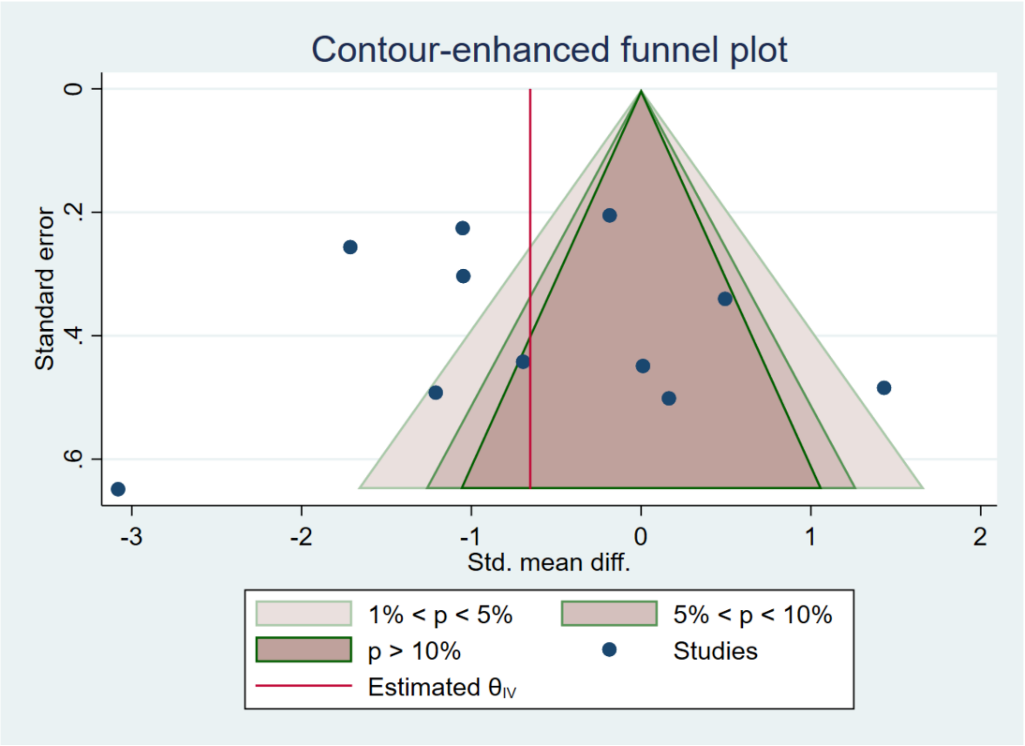

Supplementary figure S12. Funnel plot Chao1 index

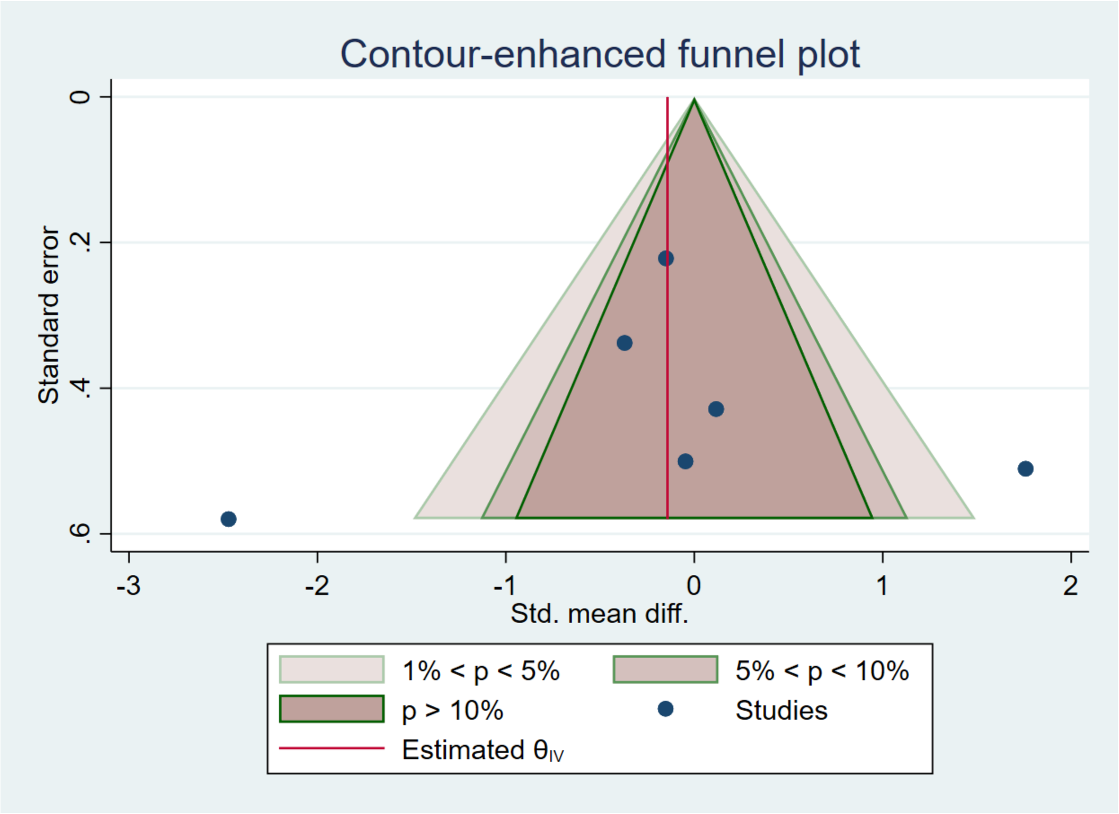

Supplementary figure S13. Risk of bias assessment

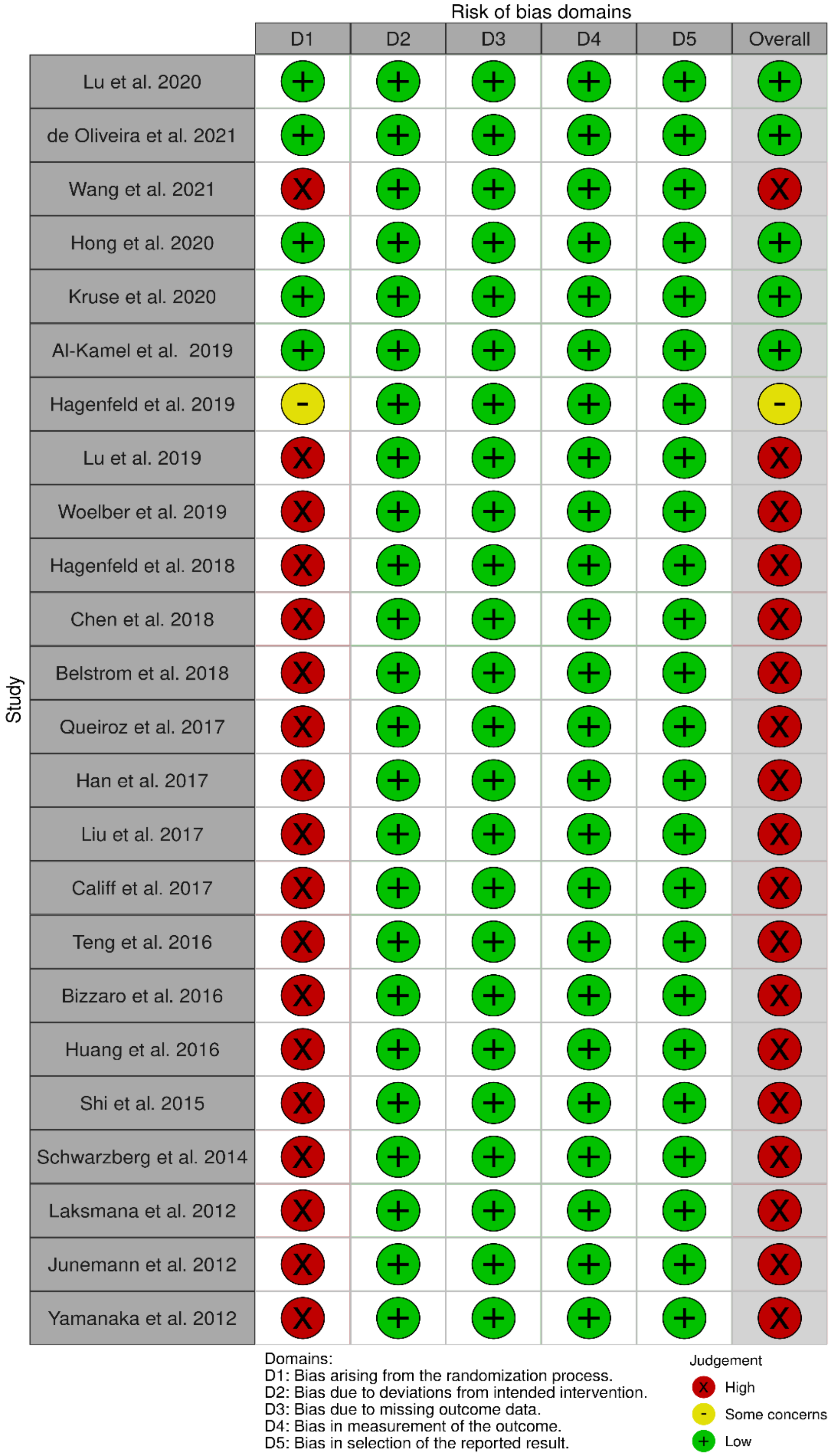

Supplementary figure S14: GRADE assessment

Question: Any form of periodontal intervention compared to no periodontal intervention/placebo for changes in alpha diversity

**Certainty assessment**							**№ of patients**		**Effect**		**Certainty**	**Importance**
**№ of studies**	**Study design**	**Risk of bias**	**Inconsistency**	**Indirectness**	**Imprecision**	**Other considerations**	**any form of periodontal intervention**	**no periodontal intervention/placebo**	**Relative (95% CI)**	**Absolute (95% CI)**		
**Richness treatment v. control (follow-up: range 1 month to 3 months)**												
8	randomized trials	serious	serious	not serious	not serious	publication bias strongly suspected	143	158	-	SMD **0.5 SD lower** (1.52 lower to 0.53 higher)	⨁◯◯◯ Very low	IMPORTANT
**Shannon index treatment v. control (follow-up: range 1 month to 12 months)**												
10	randomized trials	serious	serious	not serious	not serious	publication bias strongly suspected	199	199	-	SMD **0.6 SD lower** (1.26 lower to 0.05 higher)	⨁◯◯◯ Very low	IMPORTANT
**Chao index (follow-up: range 1 month to 3 months)**												

Certainty assessment							№ of patients		Effect		Certainty	Importance
**№ of studies**	**Study design**	**Risk of bias**	**Inconsistency**	**Indirectness**	**Imprecision**	**Other considerations**	**any form of periodontal intervention**	**no periodontal intervention/placebo**	**Relative (95% CI)**	**Absolute (95% CI)**		
6	randomized trials	serious	serious	not serious	not serious	publication bias strongly suspected	34	34	-	SMD **0.18 SD lower** (1.45 lower to 1.09 higher)	⨁◯◯◯ Very low	IMPORTANT

CI: confidence interval; **SMD:** standardized mean difference

CI: confidence interval; **SMD:** standardized mean difference
